# Promoting Walking in Cardiopulmonary Disease With Mindful Steps: Pilot Feasibility Randomized Controlled Trial of a Web-Based, Pedometer-Mediated Mind-Body Intervention

**DOI:** 10.2196/74118

**Published:** 2025-10-28

**Authors:** Kristen M Kraemer, Daniel Litrownik, Peter M Wayne, Caroline R Richardson, Neha Bhomia, Reema Kadri, Pamela M Rist, Long Ngo, Marilyn L Moy, Gloria Y Yeh

**Affiliations:** 1 Division of General Medicine, Department of Medicine, Beth Israel Deaconess Medical Center/Harvard Medical School Boston, MA United States; 2 Osher Center for Integrative Health Harvard Medical School and Brigham and Women's Hospital Boston, MA United States; 3 Warren Alpert Medical School Brown University Providence, RI United States; 4 Department of Family Medicine University of Michigan Ann Arbor, MI United States; 5 Veterans Administration Boston Healthcare System Boston, MA United States; 6 Harvard Medical School Boston, MA United States

**Keywords:** heart failure, chronic obstructive pulmonary disease, physical activity, mind-body, step counts

## Abstract

**Background:**

Physical inactivity is highly prevalent in heart failure (HF) and chronic obstructive pulmonary disease (COPD) and is associated with poor outcomes, including worsened quality of life, increased hospitalizations, readmissions, and mortality. Accessible interventions that improve physical activity are needed. Mind-body strategies are well-suited for promoting physical activity; they show promise for targeting key health behavior change processes.

**Objective:**

The aim of this study was to examine the feasibility and acceptability of a web-based pedometer-mediated mind-body intervention (Mindful Steps) for promoting walking among individuals with HF and COPD.

**Methods:**

In this pilot randomized controlled trial, participants with chronic, stable HF and COPD were randomized to Mindful Steps or usual care in a 2:1 ratio. Mindful Steps is a 12-month multimodal intervention that includes a pedometer with individualized step-count goals, live mind-body exercise (MBE) classes, and a web platform with mind-body videos, motivation messages, and educational tips. Feasibility (recruitment rate, retention), intervention acceptability, and intervention adherence were the primary outcomes. Exploratory outcomes assessed at baseline, 3-, 6-, 9-, and 12-months included daily step counts, cognitive-behavioral/psychosocial measures, health-related quality of life, and self-reported physical function. Participants were enrolled in the study from April 2019 to July 2021. The study was converted to all-digital during the pandemic after March 2020.

**Results:**

Forty-one participants were randomized to Mindful Steps (n=26) or usual care (n=15). The recruitment rate was 3% (43/178), and overall study retention was 76% (31/41). In the intervention group, over 12 months, 58% (15/26) met a predefined benchmark for MBE class adherence (attending >70% of classes). Participants engaged most consistently with the MBE classes, the pedometer, and mind-body videos. There was a positive signal regarding group differences in the change in daily step counts from baseline, favoring intervention at 3 months (estimate=2038.77 steps per day between groups, 95% CI 289.76-3788.77), 6 months (estimate=3031.45, 95% CI 1261.15-4801.74), and 9 months (estimate=2703.80, 95% CI 862.97-4544.62). There were also positive signals regarding group differences in the change from baseline favoring intervention in the following outcomes: emotional awareness (estimate=0.88, 95% CI 0.15-1.61) and body listening (estimate=1.16, 95% CI 0.25-2.07) at 3-months; internal motivation (estimate=1.03, 95% CI 0.01-2.04) and pressure/tension at 6-months (estimate=–1.59, 95% CI –2.55 to –0.63); and exercise self-efficacy at 12 months (estimate=1.77, 95% CI 0.20-3.33).

**Conclusions:**

Mindful Steps was largely feasible, acceptable, and had adequate intervention engagement. There were positive signals favoring the multimodal web intervention for daily step counts, interoceptive awareness, internal motivation, and exercise self-efficacy that will inform hypotheses in future studies. A pivot to fully remote conduct during the pandemic was successful. A larger trial examining the efficacy of Mindful Steps for promoting physical activity is warranted.

**Trial Registration:**

ClinicalTrials.gov NCT03003780; https://clinicaltrials.gov/study/NCT03003780

## Introduction

Chronic obstructive pulmonary disease (COPD) and heart failure (HF) are chronic cardiopulmonary conditions that share similar morbidity and clinically relevant decreases in physical activity. Physical inactivity is highly prevalent among individuals with COPD and HF [1-5] and is associated with lower health-related quality of life (HRQL) [6,7], increased exacerbations [8], more frequent hospitalizations and readmissions [8-11], and increased mortality [9,12-16]. In COPD, specifically, physical activity was shown to be the strongest predictor of mortality [15]. Thus, physical activity monitoring and promotion are key treatment goals for both COPD and HF [17,18]. Conventional pulmonary and cardiac rehabilitation programs include supervised aerobic exercise training, often targeting moderate intensity exercise on treadmill or bicycle ergometer, that have been shown to reduce symptoms, improve HRQL, and increase exercise capacity [19-21] However, these programs are vastly underused [22,23] among individuals with COPD or HF and may not lead to significant improvements in long-term habitual physical activity in one’s daily life outside of the formal rehabilitation setting [24-26]. While increasing regular physical activity is recommended, there is no consensus on optimal exercise modalities or regimens in COPD or HF, and studies support the continued adoption of general physical activity guidelines (including daily walking) in these populations [27,28]. There remains a critical need for highly accessible interventions that target processes relevant to sustaining daily physical activity for individuals with COPD and HF [29].

Moy et al [30,31] developed an accessible, web-based, pedometer-mediated intervention to promote walking behaviors among individuals with COPD called Every Steps Count (ESC) [30-37]. The 6-month intervention, based on self-regulation theories of health behavior change [38], included a web platform coupled with a pedometer, which provided individualized step-count goals, iterative feedback graphically and on the face of the pedometer, educational tips for disease self-management, motivational messages to help sustain walking, and a web-based community forum for social support. The intervention was safe, engaging, and associated with improvements in daily step counts and HRQL at 4 or 6 months compared with controls [30-33]. However, daily step count and HRQL improvements were not maintained at the 12-month follow-up [32]. Mindful Steps was thus designed to incorporate mind-body training as an adaptation of this original web-based intervention to further bolster and better sustain effects on walking.

Rationale, design, and conceptual model of the Mindful Steps intervention are described elsewhere [39]. In brief, Mindful Steps was developed to promote long-term walking behaviors by incorporating mind-body principles and content within the web-based, pedometer-mediated intervention. Mind-body components, such as mindful attention, breathing awareness/regulation, body awareness, and mindful movement, may support long-term physical activity by targeting key processes relevant to the initiation and maintenance of health behavior change. Indeed, mindfulness interventions may impact health behavior initiation and maintenance via changes in self-regulatory processes, such as emotion regulation, attention/cognitive control, self-related processes (eg, self-efficacy and interoceptive awareness), and motivation [40]. Mindful movement interventions, in particular (eg, tai chi and yoga), may serve as a gentle introduction to physical activity and simultaneously target processes needed to maintain physical activity, including improved physical function [41], exercise self-efficacy [42], enhanced body and self-awareness [43], and positive affective experiences during exercise [44-46]. Indeed, compared with nonmindful exercise, mindful exercise is associated with greater positive affect and enjoyment during exercise [44,47], which may promote continued physical activity engagement [48]. With the goal of targeting multiple pathways for behavior change, Mindful Steps combined adapted components from the ESC intervention (iterative feedback and goal-setting, educational and motivational messages, social support) together with new mind-body exercise (MBE) classes and a video library focused on mindful body awareness and movement, and cultivating intrinsic motivation and self-efficacy for walking. Moreover, Mindful Steps was expanded with additional educational content to be relevant for individuals with HF, in addition to COPD, given the similarities in patient phenotype, shared pathophysiology and symptoms, and need for physical activity intervention. While the ESC intervention did not include live content (ie, all components were asynchronous), we included live MBE classes to foster modeling; individuals may develop confidence and motivation from seeing others succeed (Litrownik et al [39] provides details of our theoretical model).

The aim of this pilot randomized controlled trial was to examine the feasibility and acceptability of Mindful Steps among individuals with COPD and HF and explore preliminary effect estimates on physical activity objectively measured as daily step counts, disease-specific HRQL, cognitive and psychosocial outcomes, and self-reported physical function compared with usual care. In line with guidelines for pilot trials [49], findings from this study will inform the design of a fully-powered RCT to examine the efficacy of Mindful Steps for promoting and sustaining long-term adherence to walking in COPD and HF.

## Methods

### Study Design

Individuals with COPD or HF were randomized in a 2:1 ratio to a 12-month Mindful Steps intervention or usual care. Following the CONSORT (Consolidated Standards of Reporting Trials) guidelines for pilot trials [50], main outcomes included study feasibility and acceptability. Exploratory outcomes were included to inform hypotheses and the design of a future larger-scale RCT. Participants were assessed at baseline, 3, 6, 9, and 12 months. Study staff completing assessments were blinded to group assignment. Details of the study protocol are published [39] and briefly summarized below.

### Ethical Considerations

The study was approved by the Beth Israel Deaconess Medical Center Institutional Review Board (IRB # 2016P000368), and all participants provided written informed consent. Study data were stored confidentially using REDCap (Research Electronic Data Capture; Vanderbilt University), which is Health Insurance Portability and Accountability Act**–**compliant, password-protected, and managed by Beth Israel Deaconess Medical Center, or stored on secure institutional servers. Participant privacy was maintained at all times. All participants were assigned a study ID. Participants received up to US $200 via check for study participation and kept their Fitbit devices (approximately US $80 in value).

### Recruitment, Eligibility, and Randomization

Potential participants, identified through hospital registries, physician clinic schedules (cardiology and pulmonology), and specialist and primary care physician referrals, were sent an opt-out mailing and contacted by telephone by research assistants from February 2019 to May 2020. Participants were also recruited via flyers, digital screen ads in specialist and primary care clinic waiting rooms, and in-person presentations at local cardiac or pulmonary rehabilitation programs (for those who completed or no longer engaged in the program). Interested individuals completed a phone screen and scheduled an in-person consent visit.

Individuals were eligible if they met the following criteria: (1) older than 40 years; (2) clinical diagnosis of COPD, defined as either a ratio of forced expiratory volume in one second to forced vital capacity <0.70 or chest computed tomography evidence of emphysema, or clinical diagnosis of HF syndrome (with left ventricular systolic dysfunction or preserved ejection fraction, and New York Heart Association Class 1-3); (3) medical clearance from a provider to participate in an exercise program; (4) an active email account with the ability to check email at least weekly; and (5) access to a computer with an internet connection, USB port, and Windows. Individuals were excluded if they met any of the following criteria: (1) self-reported COPD or HF exacerbation in the previous 2 weeks, (2) inability to ambulate, (3) clinical signs of unstable cardiovascular disease, (4) hypoxemia during 6-minute walk test (oxygen saturation <85% with supplemental oxygen), (5) inability to collect at least 7 of 14 days of baseline step counts, and (6) current participation in a cardiac or pulmonary rehabilitation program.

Participants completed a 14-day run-in period where they were asked to wear the pedometer (Fitbit Alta HR; Fitbit Inc) all waking hours. Seven days of valid data (>200 steps per day) were required for randomization. Participants were then randomized in a 2:1 ratio to Mindful Steps or usual care using block randomization with varying/unpredictable block sizes. The random numbers were computer-generated and concealed by sealed and numbered opaque envelopes. After eligibility was confirmed, research assistants opened the next envelope in sequence and informed the participant of their assignment. The study was conducted in 4 waves (cohorts of ≈10 participants each). During the COVID-19 pandemic, all study activities, including recruitment, consent, and testing, were transitioned to all-remote procedures in March 2020.

### Interventions

All participants continued to receive pharmacological treatment and usual care from their health care providers. In addition, all participants received an education booklet from Harvard Health Publishing titled “Walking for Health,” which describes the health benefits of walking, strategies for starting a walking program, and specific walking workouts [51].

### Mindful Steps

This was a 12-month multicomponent web-based, pedometer-mediated intervention that included: (1) a pedometer (Fitbit Alta HR); (2) the Mindful Steps web platform with mind-body videos, motivational messages, educational content, a web-based community forum; and (3) live MBE classes.

The pedometer is paired with the Mindful Steps web platform to visually display the participant’s daily/weekly steps and individualized step goals. Participants were asked to upload their step counts to the study server at least once a week. The initial step-count goal was calculated using an algorithm designed to promote slow and safe increases, where the most recent 7 consecutive days of synced data were averaged and 400 steps were added. Previous research among individuals with type 2 diabetes used an 800-step increment based on participant feedback [52]. Subsequent research selected a 400-step increment in COPD as a more attainable goal in this population [31]. Subsequent goals could increase, decrease, or stay the same, but not go up by more than 400 steps over the previous goal and not exceed 10,000 steps for safety reasons. In the absence of new data, the prior week’s goal was reissued. Participants were asked to wear the wrist-worn pedometer daily during waking hours. Motivational messages and educational tips focused on self-managing HF and COPD and walking-related topics (eg, “Managing Stress and Anxiety,” “Handling Setbacks,” and “Preparing for Your Walk”). A new motivational message appeared on the web homepage weekly throughout the 12-month intervention (a total of 52) and a new educational tip every other day for the first 6 months (a total of 88; repeated in the final 6 months). Education content also embedded additional disease-specific resource links (eg, American Heart Association, Heart Failure Society of America, and COPD Foundation). The web-based community forum allowed participants to post comments or respond to seeded posts from study staff to stimulate discussion (eg, “Share something new you noticed on your walk today”); participants were able to respond to other participants’ posts in these threads. To help promote interaction with the study website, the web platform included a visual star reward system. Participants would earn up to 3 stars daily for reaching their step-count goal, watching videos, and viewing the motivational messages and educational tips. Stars were retroactively awarded for meeting step-count goals even if participants did not upload their steps daily.

The Mindful Steps web platform also included 2 sets of mind-body videos that participants could access, which were designed to support and integrate with the live MBE classes (described below). The first set was a themed mindful walking video curriculum that included 26 videos mirroring the themes from the live MBE classes. A new mindful walking video was posted on the web homepage weekly for the first 6 months (repeated in the second 6 months). Each video included a brief didactic introduction to the theme and a guided MBE related to the theme. The second set was an MBE video library, including 13 videos demonstrating specific exercises taught in classes to aid in home practice. Participants were encouraged to use the website daily and watch the weekly mindful walking video.

Live MBE classes were 75 minutes and held weekly during the first 6 weeks of the intervention, biweekly for the next 34 weeks, and monthly for the last 12 weeks. Each class focused on a theme for promoting walking and included a guided meditative walk and a menu of MBEs used in our previous trials [53-56]. MBEs included mindful warm-up and stretching exercises, and traditional qigong movement and breathing exercises [39]. In-person MBE classes were transitioned to live videoconferencing via Starleaf (the hospital’s preferred meeting platform in 2020) during the COVID-19 pandemic in March 2020. Classes were scheduled at a time that was convenient for the group during regular business hours. There were opportunities for real-time interaction with instructors and other participants during the classes on videoconference.

The themes of the mind-body exercise classes and mindful walking video curriculum in the Mindful Steps intervention were (1) introduction: body, mind, and breath; (2) mindful warm-ups: lower body; (3) motivation to move; (4) putting joy back into exercise; (5) rewarding yourself with the gift of walking; (6) mindfulness in motion; (7) mindful warm-ups: upper body; (8) renew your body with your breath; (9) every step counts; (10) self-kindness; (11) overcoming barriers and challenges; (12) walking for your mind and spirit; (13) pain management; (14) preventing falls; (15) strength and flexibility; (16) breath awareness; (17) your body affects your mind; (18) stop and smell the roses; (19) relaxation and stress management; (20) exploring balance in walking; (21) belly breathing; (22) rest and recharge along the way; (23) importance of posture; (24) leg strength; (25) moderated effort: the 70% rule; and (26) mechanics of walking.

### Outcome Measures

#### Recruitment and Retention

Study feasibility was assessed via recruitment and retention rates. The a priori benchmark for successful retention was 80%, or less than 20% dropouts.

#### Intervention Acceptability and Adherence

Acceptability was primarily assessed via qualitative exit interviews at 6 and 12 months. The qualitative results are published elsewhere [57]. In addition, participants were asked to rate weekly, using a sliding scale, the likeability (0=dislike to 100=really like) and helpfulness with walking (0=not at all to 100=very much) of each of the 26 videos in the mindful walking video curriculum. Average likeability and helpfulness ratings were calculated using the ratings obtained from the first 26 weeks.

Intervention adherence included MBE class adherence, engagement with the web platform, and use of the pedometer. The a priori definition of adherence to MBE classes was 70% attendance (17 of 25 classes). Home practice included practicing exercises from the video library or live classes and was tracked via weekly self-report surveys, including the number of minutes of practice, if any. Web platform use was automatically tracked and included the total number of pages visited within the website (ie, total of each individual click on a page on the website), total number of stars earned, and total/unique clicks on pages with educational tips and motivational messages. Video watch time was assessed via the number of minutes each participant watched any of the videos. Pedometer use was assessed via the total number of days with any step counts greater than 0.

#### Safety

Adverse events were systematically assessed at 3-, 6-, 9-, and 12-month testing visits. Participants were asked about specific symptoms, including muscle strain/soreness, dizziness, episodes of shortness of breath, falls, chest discomfort, and any other adverse events not specifically queried. Participants were also asked about emergency room visits and hospitalizations. In addition, participants were sent weekly surveys that asked them to report any symptoms or adverse events.

### Exploratory Outcomes

#### Physical Activity

##### Daily Step Counts

Average daily step counts were measured by a Fitbit Alta HR monitor worn on the wrist. At each study visit, participants in usual care were asked to wear the pedometer for 14 days during waking hours except when showering/bathing. Similarly, those in the intervention group continued to wear the pedometer as usual. At each time point, data from 14 days starting at the testing visit were extracted for analysis. A wear day was considered valid if ≥200 steps were recorded. An average daily step count variable was created by averaging daily step counts across all valid wear days.

##### Community Healthy Activities Model Program for Seniors Physical Activity Questionnaire

The Community Healthy Activities Model Program for Seniors Physical Activity Questionnaire [58] is a 41-item self-report measure that assesses the frequency and duration of multiple activities across differing exercise intensities among older adults. The Community Healthy Activities Model Program for Seniors Physical Activity Questionnaire allows the calculation of weekly frequency and estimation of weekly caloric expenditure, which we calculated for both “all activity” and “moderate intensity activity.”

### Disease-Specific HRQL

#### Minnesota Living With Heart Failure Questionnaire

The Minnesota Living With Heart Failure Questionnaire [59] is a 21-item self-report measure assessing HRQL among individuals with HF. Items are rated on a 6-point Likert scale (0=no to 5=very much). This instrument was used only in study participants with HF.

#### St George’s Respiratory Questionnaire

The St George’s Respiratory Questionnaire [60] is a 50-item measure that assesses HRQL among individuals with respiratory illnesses. Total scores range from 0 to 100, with lower scores reflecting better HRQL. This instrument was used only in study participants with COPD.

### Cognitive-Behavioral and Psychosocial

#### Self-Efficacy for Exercise Scale

The Self-Efficacy for Exercise Scale [61] is a 9-item self-report measure that assesses one’s confidence for exercise in the face of barriers (eg, when feeling tired). Items are rated on a scale from 0 (not at all confident) to 10 (very confident).

#### Self-Efficacy for Managing Chronic Disease Scale

The Self-Efficacy for Managing Chronic Disease Scale [62] is a 6-item self-report measure that assesses one’s confidence in managing their chronic illness. Items are rated on a scale from 1 (not at all confident) to 10 (totally confident).

#### Intrinsic Motivation Inventory

The Intrinsic Motivation Inventory (IMI) [63] is a self-report measure that assesses motivation for a specific activity. The 22-item version was used in this study, and the items were modified to assess the activity of walking. The 22-item version has 4 subscales: interest/enjoyment, perceived competence, perceived choice, and pressure/tension. Items are rated on a 7-point Likert scale (1=not at all to 7=very true).

#### Patient Activation Measure

The PAM [64] is a 13-item self-report measure that assesses one’s skills, knowledge, and confidence for managing their chronic illness. Items are rated on a scale from 1 (strongly disagree) to 4 (agree strongly), and raw scores are converted into an activation score ranging from 0 to 100, with higher scores reflecting greater activation.

#### Medical Outcomes Study Social Support Survey

The Medical Outcomes Study Social Support Survey [65] is a 19-item self-report measure that assesses social support among individuals with chronic illnesses. Items are rated on 5-point Likert scale (1= none of the time to 5= all of the time). The scale includes a total score and 4 subscales assessing specific types of support: emotional/informational support, tangible support, affectionate support, and positive social interaction.

#### Multidimensional Assessment of Interoceptive Awareness

The Multidimensional Assessment of Interoceptive Awareness (MAIA) [66] is a 32-item self-report measure that assesses interoceptive body awareness. Items are rated 6-point Likert scale (0=never to 5=always). The 8 separately scored scales of the MAIA include: noticing, not-distracting, not-worrying, attention regulation, emotional awareness, self-regulation, body listening, and body trusting.

#### Center for Epidemiologic Studies Depression Scale

The Center for Epidemiologic Studies Depression Scale [67] is a 20-item self-report measure assessing symptoms of depression. Items are rated on a scale from 0 (rarely or none of the time) to 3 (most or almost all the time).

### Physical Function

Physical function (10-item measure), fatigue (7-item measure), and pain interference (8-item measure) were assessed using the National Institutes of Health PROMIS (Patient-Reported Outcomes Measurement Information System) scales [68-71]. Higher scores reflect better physical function, greater fatigue, and more pain interference. Scores are calculated using a standardized T-score system with a population mean of 50 (SD 10).

All survey instruments were initially administered at in-person testing visits; subsequently, they were administered over Zoom or phone once the study transitioned to remote study procedures during the COVID-19 pandemic.

### Statistical Analysis

#### Feasibility, Acceptability, Intervention Adherence, and Safety

Feasibility and adherence, including website engagement, were assessed using descriptive statistics, including means, SDs, medians, IQRs, and frequencies. For adherence, descriptive statistics were calculated for the first 6 months, the final 6 months, and the total 12 months of the intervention to assess patterns over time. Given that website usage data were objectively tracked (eg, clicks and video watch time), missing data are an absence of website engagement and therefore were entered as zero. Acceptability of the study videos was assessed by calculating average likeability and helpfulness ratings for each of the 26 videos and reporting the range of these ratings. Safety was assessed via the number of adverse events according to expectedness, relatedness to the study, and severity.

#### Preliminary Effect Estimates

In line with guidelines for pilot trials [72,73], we assessed changes in patient-centered outcomes, including cognitive-emotional constructs from our theoretical model [39], to explore which measures may be sensitive to change and inform variability within and between groups to inform the design of future studies. For each patient-centered outcome, we computed the difference (between treatment arms) of differences (from baseline). We performed repeated measures analyses using the linear mixed effects models (SAS PROC MIXED) to evaluate trajectories over the course of the 4 time points for each group [74-76]. The estimated mean differences between the groups in these trajectories at each time point were obtained via interaction terms (treatment group × time) and linear contrasts. Cohen *d* effect sizes were computed to evaluate the magnitude of group differences in change from baseline.

## Results

### Recruitment and Retention

**Figure 1:**
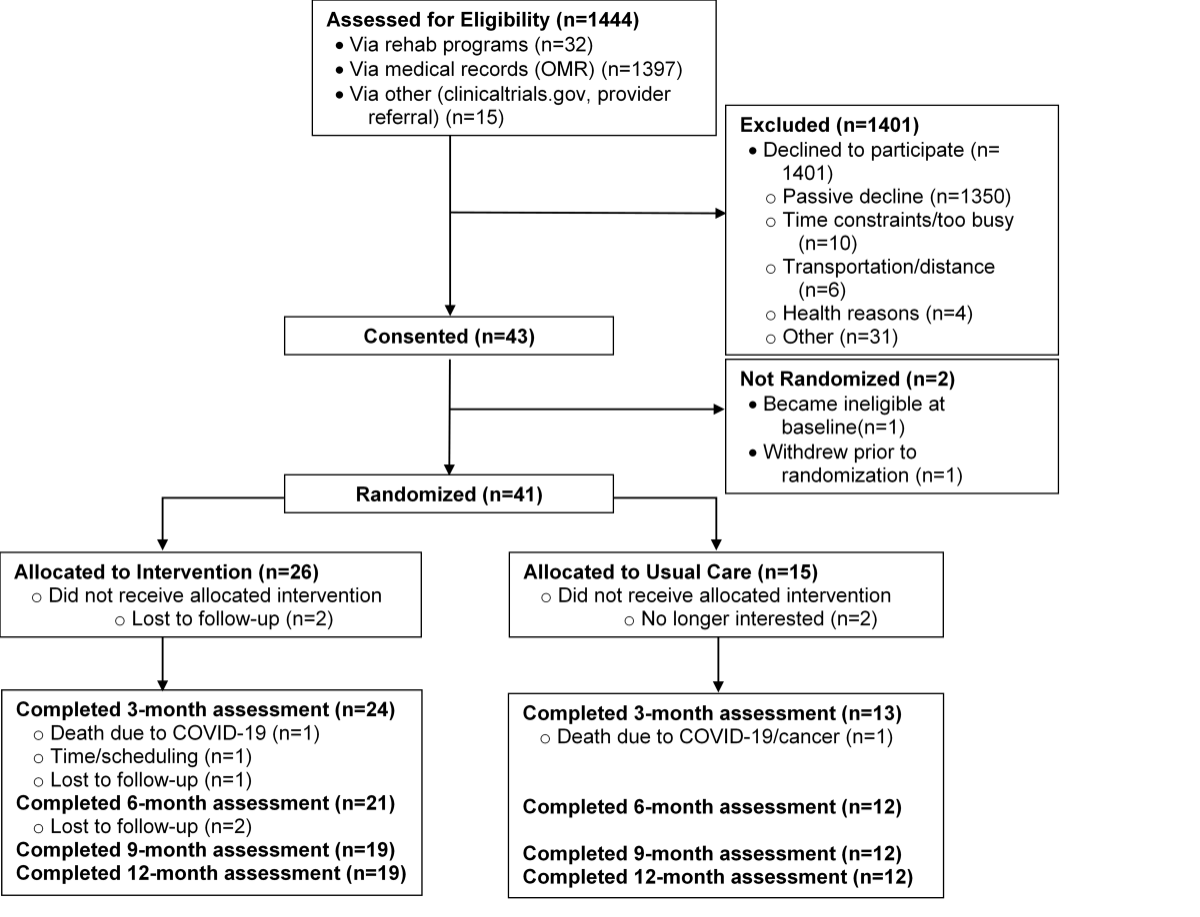
CONSORT (Consolidated Standards of Reporting Trials) flow diagram of recruitment, randomization, and retention in the Mindful Steps pilot randomized controlled trial among individuals with chronic obstructive pulmonary disease or heart failure.

Recruitment and retention are detailed in the CONSORT diagram (Figure 1). Of 1444 individuals (reached out to via letter or in-person at rehabilitation program), we made further contact with 178 to assess interest and eligibility. Of those, 43 consented to participate, for an overall recruitment rate of 3%. Forty-one participants were randomized, and 31 completed the 12-month study (retention rate=76%). Of those randomized who did not complete the study, 1 dropped out due to time/scheduling, 2 were no longer interested in the study, 2 were deceased (1 due to COVID, 1 due to COVID-19 and metastatic cancer; both unrelated to the study), and 5 dropped out due to “other reasons.”

### Baseline Characteristics

Baseline characteristics are summarized in Table 1. Participants were 56% (23/41) female with a mean age of 69.99 (SD 8.0) years. For qualifying diagnoses, 56% (23/41) of participants had COPD, 34% (14/41) had HF, and 10% (4/41) had a diagnosis of both COPD and HF. Most participants (36/41, 88%) self-identified as White, 7% (3/41) Black, 2% (1/41) Asian, and 2% (1/41) other. Regarding rehabilitation status, 22% (9/41) had previously completed pulmonary rehabilitation, and 7% (3/41) cardiac rehabilitation. The mean Charlson Comorbidity Index was 4.95 (SD 1.75), indicating fairly high morbidity in our sample (Charlson Comorbidity Index score 3-4=moderate, ≥5=severe [77]). The mean tobacco pack-years was 34.09 (SD 28.56).

**Table 1:** Baseline sociodemographic and clinical characteristics of study participants with chronic obstructive pulmonary disease and heart failure in the Mindful Steps pilot randomized controlled trial.

Characteristic				Intervention (n=26)		Control (n=15)		Total sample (N=41)
Age (years), mean (SD)				69.88 (8.1)		70.17 (8.1)		69.99 (8.0)
Sex (female), n (%)				14 (54)		9 (60)		23 (56)
**Race, n (%)**								
		White	22 (85)		14 (93)		36 (88)	
		Black	2 (8)		1 (7)		3 (7)	
		Asian	1 (4)		0 (0)		1 (2)	
		Other	1 (4)		0 (0)		1 (2)	
Annual income <US $35,000, n (%)				4 (15)		2 (13)		6 (15)
Married, n (%)				18 (69)		11 (73)		29 (71)
Unemployed, retired, or disabled, n (%)				22 (85)		7 (47)		29 (71)
**Qualifying diagnosis, n (%)**								
		COPD	14 (54)		9 (60)		23 (56)	
		Chronic heart failure	8 (31)		6 (40)		14 (34)	
		Both	4 (15)		0 (0)		4 (10)	
Charlson Comorbidity Index, mean (SD)				4.92 (1.81)		5.00 (1.69)		4.95 (1.75)
Regular oxygen use, n (%)				2 (8)		1 (7)		3 (7)
Smoking pack years, mean (SD)				32.06 (30.98)		37.96 (24.34)		34.09 (28.56)
Completed pulmonary rehabilitation, n (%)				8 (31)		1 (7)		9 (22)
Completed cardiac rehabilitation				2 (7.7)		1 (7)		3 (7)
**Comorbidities, n (%)**								
	CVD^a^ (CAD^b^, Angina, Angioplasty, and MI^c^)		3 (12)		2 (13)		5 (12)	
	Heart failure		12 (46)		6 (40)		18 (44)	
	Cancer		4 (15)		2 (13)		6 (15)	
	Hypertension		18 (69)		11 (73)		29 (71)	
	Limitation of limb (weakness, paralysis)		2 (8)		2 (13)		4 (10)	
	OA^d^, sciatica, chronic back pain		16 (62)		9 (60)		25 (61)	
	Peripheral vascular disease		0 (0)		1 (7)		1 (2)	
	Stroke or cerebrovascular disease		1 (4)		0 (0)		1 (2)	
	Connective tissue disease		1 (4)		1 (7)		2 (5)	
		Obstructive sleep apnea	8 (31)		4 (27)		12 (29)	
		Kidney disease	3 (12)		4 (27)		7 (10)	

^a^CVD: cardiovascular disease.

^b^CAD: coronary artery disease.

^c^MI: myocardial infarction.

^d^OA: osteoarthritis.

### Intervention Acceptability and Adherence

Among participants who completed ratings (n range=15-22), average likeability ratings for the 26 weekly videos of the mindful walking curriculum ranged from 68.59 (SD 23.14) to 80.19 (SD 8.42), and average helpfulness for daily walking ratings ranged from 58.23 (SD 29.52) to 77.06 (SD 16.08).

In terms of intervention adherence, among those randomized to Mindful Steps, 58% of participants (15/26) attended 17 or more of the live MBE classes. Of those who completed the study 79% (15/19) attended 17 or more of the live MBE classes. The first 2 cohorts of intervention participants (cohort 1 n=4, cohort 2 n=7) had a “hybrid” experience with in-person classes early during the 12-month study period before converting to digital classes after the start of the COVID-19 pandemic (first cohort=92%, 23/25 in-person classes; second cohort=52%, 13/25 in-person classes). The third and fourth cohorts (cohort 3: n=8; cohort 4: n=7) had all MBE classes conducted via videoconferencing. There were no significant differences in class attendance in-person or via web.

Table 2 presents home practice adherence in the first 6 months, the second 6 months, and the full 12 months of the study. Across 12 months, 22 participants completed home practice forms and reported “yes” to home practice in 32.05 (SD 19.92) weeks (range 1-52 weeks) and completed an average of 48.54 (SD 30.81) minutes of practice per week. The mean number of Fitbit wear days in the Mindful Steps group was 294.65 (SD 109.71) days of an expected maximum of 365 days.

**Table 2:** Home practice of mind-body exercise completion of participants with chronic obstructive pulmonary disease and heart failure, randomized to the Mindful Steps intervention.

	First 6 months (n=22)			Second 6 months (n=17)			Full 12 months (n=22)	
	Mean (SD)	Range	Mean (SD)		Range	Mean (SD)		Range
Total weeks reporting home practice	17.77 (9.29)	1-26	18.47 (9.17)		0-26	32.05 (19.92)		1-52
Home practice time (mins/week)	45.45 (27.43)	12.75-127.50	54.39 (39.92)		11.96-129.56	48.54 (30.81)		13-127.50

Website usage for the first 6 months, the second 6 months, and the total 12 months of the intervention is summarized in Table 3. For the total 12 months, participants (n=25) watched a mean 20.28 (SD 14.30; median 18.00, IQR 5.00-34.50) unique videos, with a mean 335.76 minutes (SD 515.35; median 103.40, IQR 27.31-413.55) of video watch time. Participants earned a mean of 295.64 (SD 281.45; median 214.00, IQR 104.50-411.00) stars throughout the 12 months. On average, 69% of stars were earned through reaching step goals, 17% from watching videos, and 14% from other content clicks on the website. The mean number of website pages visited throughout the 12 months was 1220.68 (SD 1400.53; median 563.00, IQR 203.50-2193.50). Throughout the 12-month intervention, participants visited a unique tip website page a mean of 22.80 (SD 28.21; median 10.00, IQR 0.50-39.00) times and a unique motivational message page a mean of 19.36 (SD 19.59; median 12.00, IQR 2.50-40.00) times. Ten participants (38%) made at least one comment in the web-based forum.

**Table 3:** Engagement with the Mindful Steps website components in a pilot randomized controlled trial among individuals with chronic obstructive pulmonary disease or heart failure.

	First 6 months (n=25)^a^				Second 6 months (n=25)^a^				Full 12 months (n=25)^a^		
	Mean (SD)	Median (IQR)	Range	Mean (SD)		Median (IQR)	Range	Mean (SD)		Median (IQR)	Range
Unique videos watched	18.48 (13.58)	15.00 (5.00-30.50)	0-39	11.04 (11.36)		5.00 (0.00-23.50)	0-30	20.28 (14.30)		18.00 (5.00-24.50)	0-40
Video watch time (mins)	200.30 (274.01)	91.45 (22.89-239.80)	0-1140	134.69 (250.66)		21.90 (0.00-138.51)	0-1103	335.76 (515.35)		103.40 (27.31-413.55)	0-2244
Stars earned	163.76 (138.28)	113.00 (70.00-231.00)	3-489	131.88 (148.23)		88.00 (24.50-173.50)	0-545	295.64 (281.45)		214.00 (104.50-411.00)	3-979
Nonunique clicks: any website page	726.96 (790.32)	376.00 (16.00-1464.50)	5-2484	488.40 (679.11)		215.00 (0.00-738.50)	0-2696	1220.68 (1400.53)		563.00 (203.50-2193.50)	5-4769
Unique clicks: educational tips	21.56 (27.85)	9.00 (0.50-36.50)	0-85	2.84 (4.06)		0.00 (0.00-5.00)	0-13	22.80 (28.21)		10.00 (0.50-39.00)	0-85
Unique clicks: motivational messages	10.80 (10.83)	6.00 (2.50-18.50)	0-35	10.32 (13.01)		2.00 (0.00-24.50)	0-41	19.36 (19.59)		12.00 (2.50-40.00)	0-52
Nonunique clicks: educational tips	38.52 (55.39)	12.00 (0.50-64.00)	0-159	3.60 (5.34)		0.00 (0.00-6.50)	0-15	42.48 (59.86)		13.00 (0.50-73.50)	0-177
Nonunique clicks: motivational messages	29.20 (36.90)	11.00 (2.50-63.00)	0-109	47.04 (78.74)		9.00 (0.00-58.00)	0-239	76.76 (110.67)		20.00 (2.50-108.50)	0-317

^a^Missing data are the absence of website engagement and therefore were entered as 0.

### Adverse Events

One adverse event was considered reportable per the BIDMC Institutional Review Board, which was deemed serious and possibly related and included an episode of noncardiac chest pain in an intervention participant with HF upon arrival for 6-month testing. The patient was evaluated in the emergency department and discharged with possible musculoskeletal pain and anxiety. Of 85 nonreportable events (55 in intervention, 30 in usual care), 3 were deemed expected and possibly related, including mild-moderate musculoskeletal pain during walking or MBE classes in the intervention group.

### Preliminary Effect Estimates

**Figure 2:**
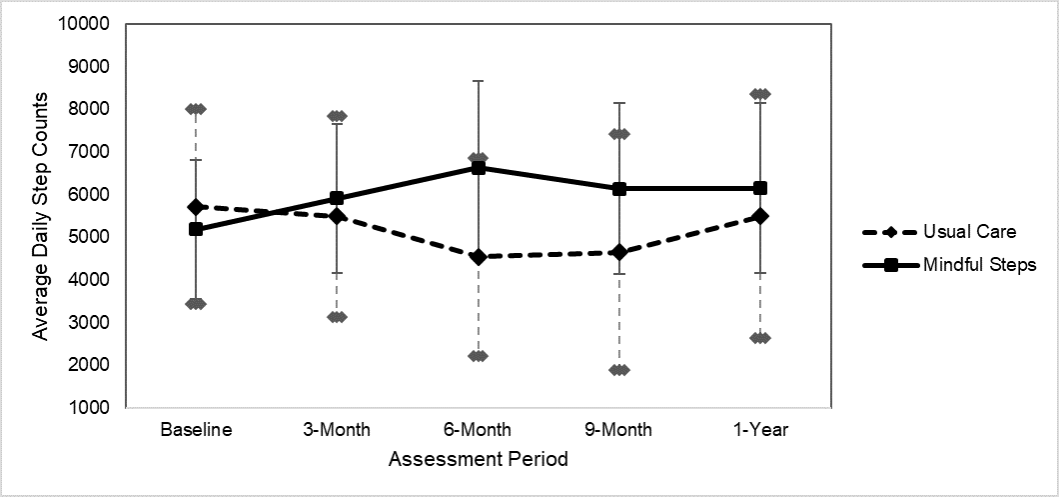
Mean daily step counts with 95% CI at each assessment period in a pilot randomized controlled trial comparing Mindful Steps to usual care among individuals with chronic obstructive pulmonary disease or heart failure.

Results on the effects of Mindful Steps and usual care on daily step counts are presented in Table 4. Figure 2 provides a graphical display of mean daily step counts at each timepoint. In mixed effect models, there were greater increases in daily step counts from baseline to 3 months (estimate=2038.77 steps per day between groups, 95% CI 288.76-3788.78), 6 months (estimate=3031.45, 95% CI 1261.15-4801.74), and 9 months (estimate=2703.80, 95% CI 862.97-4544.62) in the Mindful Steps group compared with usual care. Effect sizes for group differences in the change from baseline to each timepoint were medium-to-large (Cohen *d* range 0.73-1.18). The difference in the change in daily step counts between groups from baseline to 12 months was not significant, although the effect size is medium-to-large (Cohen *d*=0.73).

**Table 4:** Comparison of daily step counts between groups (Mindful Steps intervention vs usual care) among individuals with chronic obstructive pulmonary disease or heart failure.

	Mindful Steps (n=26)			Usual care (n=15)			Difference in differences (95% CI)^a,b^		*P* value^b^		Cohen *d* of difference in differences
	Mean (SD)^c^	Difference from baseline, mean (SD)	Mean (SD)^c^		Difference from baseline, mean (SD)						
Baseline	5180.74 (4013.46)	0 (0)	5710.44 (4130.39)		0 (0)	—^d^		—		—	
3-month	5910.11 (4049.34)	1066.90 (2128.21)	5489.02 (3699.05)		–1074.49 (950.19)	2038.77 (288.76 to 3788.78)		.02		1.18	
6-month	6628.06 (4325.51)	1320.68 (3351.20)	4537.91 (3648.83)		–1821.20 (1508.98)	3031.45 (1261.15 to 4801.74)		<.01		1.11	
9-month	6139.62 (4162.30)	882.71 (3457.38)	4650.36 (3864.16)		–2033.88 (1999.38)	2703.80 (862.97 to 4544.62)		<.01		0.96	
12-month	6149.94 (3739.57)	714.62 (2842.02)	5485.72 (4257.90)		–1333.01 (2789.37)	1427.94 (–454.45 to 3309.33)		.13		0.73	

^a^Difference from baseline of Mindful Steps minus the difference from baseline of usual care.

^b^Linear Mixed Effects Model testing the difference of differences from baseline between Mindful Steps and usual care.

^c^Average daily step counts within a 14-day window around each timepoint.

^d^Not applicable.

Results on the effects of Mindful Steps and usual care on all other exploratory outcomes are presented in Table S1 in Multimedia Appendix 1. In mixed effect models, there were greater improvements in MAIA body listening (estimate=1.16, 95% CI 0.25-2.07) and MAIA emotional awareness (estimate=0.88, 95% CI 0.15-1.61) from baseline to 3 months in Mindful Steps compared with usual care. There were greater improvements in IMI-Interest (estimate=1.03, 95% CI 0.01-2.04) and IMI-Pressure (estimate=–1.59, 95% CI –2.55 to –0.63) from baseline to 6 months in Mindful Steps compared with usual care. There were greater improvements in exercise self-efficacy from baseline to 12 months in Mindful Steps compared with usual care (estimate=1.77, 95% CI 0.20-3.33). There were no significant group differences in the changes from baseline to any timepoint for all other outcomes.

## Discussion

The aim of this study was to assess the feasibility and acceptability of a multicomponent web-based, pedometer-mediated, mind-body intervention to improve and sustain walking among individuals with COPD or HF. Findings suggest that the study was feasible. While the recruitment rate was slower than expected, the rate is similar to other reports in this population and reflects commonly cited barriers of time and transportation [78-80]. We also noted a significant number of passive declines, which largely reflects our recruitment process involving mass mailings from a hospital database. Once randomized, more than three-quarters of participants were retained in the study at 12 months, slightly below our predefined benchmark of 80%. Recruitment and retention were additionally hindered by the COVID-19 pandemic, which occurred midway through the study. There were no significant, unexpected safety concerns.

Adherence to the 12-month Mindful Steps intervention was adequate. Participants engaged most with the MBE classes, pedometer, and mind-body videos. Over half of all participants randomized to Mindful Steps achieved our predefined benchmark of 70% class attendance; as expected, the proportion of participants achieving this benchmark was much higher when excluding those lost to follow-up. On average, participants watched more than half of the available videos on the website, with moderate to good scores for likeability and helpfulness. Participants also demonstrated good compliance with the pedometer, wearing the Fitbit on most (≈295) days in a year. Engagement with motivational messages, tips, and the web-based forum on the website was less robust. Of note, for all website engagement metrics, the mean was higher than the median, suggesting that a proportion of high website engagers may have impacted overall averages. This intervention was designed to better support walking up to 12 months, and results may suggest modest maintenance of step gains, but in this pilot feasibility study, we are unable to compare with the ESC intervention with pedometer and web-platform alone without mind-body content [32]. Nonetheless, there were still notable declines in website engagement in the second 6 months. The exception to this was clicks on motivational messages, which were similar or higher in the second compared with the first 6 months. Overall, these engagement results highlight the known challenges and overall difficulty engaging patients with COPD and HF long-term in behavioral interventions [32,81]. Future research could examine additional strategies to enhance website engagement (eg, leaderboards and the ability to comment on others’ achievements).

The intervention engagement data are consistent with our published qualitative data [57]. In individual qualitative interviews at 6 months, participants endorsed the pedometer with feedback, MBE classes, and mind-body videos as the most helpful components of the intervention. In developing this multimodal intervention, we purposefully included multiple components, which provided a multipronged approach to behavior change that targeted multiple cognitive-behavioral constructs based on our conceptual model [39]. In addition, on the web-platform alone, there were several elements to engage with, as we recognized that participant engagement with the varied content might differ based on individual preferences and needs. To further optimize the intervention, future work might use innovative designs (eg, using the Multiphase Optimization Strategy framework) to better understand the contribution of individual components, and examine whether engagement with different components predicts physical activity outcomes [82]. Factorial designs could also inform whether the mind-body content in Mindful Steps added benefit beyond the ESCs intervention. Further research might also explore the declines in website engagement in the later periods of the intervention, and also the relationship between declines in website engagement and the rate of daily step count change. It is possible that participants have built confidence in the first 6 months to practice/implement mind-body strategies on their own, only needing to return to the website intermittently to review or reinforce a skill, as step-count data support a positive effect on physical activity that is strongest at 6 months but still durable at 12 months. Alternatively, it is possible that more consistent website engagement may maximally target physical activity outcomes, but website fatigue or other barriers interfere with engagement, thus limiting the overall intervention effects. While our intention was to include a live component (ie, MBE classes) to foster modeling and connectedness, future research might examine the impact of this live component on scalability and explore additional avenues for targeting these processes.

Consistent with guidelines for pilot trials [72,73], the aim of this study was to identify outcomes sensitive to change to inform the design of a future larger trial. Importantly, daily step counts were sensitive to change in this study, and there were indeed medium-to-large between-group differences in change in step counts from baseline to 3-, 6-, and 9-months, favoring Mindful Steps. While some of the initial improvements waned in the final 6 months, there was still a relative increase from baseline to 12 months, both within the intervention group and compared with usual care. Given the pilot nature of this study, future larger trials are needed to examine the efficacy of Mindful Steps for improving daily step counts.

In addition, we found that important constructs from our theoretical model [39], including indices of interoceptive awareness, internal motivation, and exercise self-efficacy, were sensitive to change. We saw medium-to-large changes in interoceptive awareness and internal motivation in the first several months of the intervention, which were explicit intervention targets in our conceptual model. Interoceptive and body awareness are inherently cultivated in all of the mindful movements presented in Mindful Steps. One of the main recurring themes of the mindful walking curriculum and MBE classes emphasized internal motivation (eg, “Motivation to Move,” “Putting Joy Back Into Exercise,” and “Rewarding Yourself with the Gift of Walking”), which occurred early in the intervention. Thus, as expected, participants in Mindful Steps reported more motivation due to interest or enjoyment in the activity and less due to external pressures within the first 6 months. These findings are also consistent with our published qualitative data [57], where participants noted body awareness as a strategy that helped with their walking and a shift in walking enjoyment and internal motivations for walking. Interestingly, differences between groups in self-efficacy did not emerge until 12 months. It is possible that longer-term practice with mind-body strategies and walking is needed to move the needle on self-efficacy, particularly in HF and COPD populations that have longstanding fears of physical activity due to breathlessness [83].

Several study limitations are worth noting. First, as this was a small pilot study, results should be used to inform the design and hypotheses for a future larger study and should not be interpreted regarding efficacy. Second, while we intended a more even mix, we enrolled more patients with COPD than HF; however, we do not believe this impacts the overall generalizability to a chronic cardiopulmonary disease population. Given the sample size and scope of this trial, we are not able to do subgroup analyses to determine if intervention effects are different among the 2 conditions. Similarly, we did not collect information on time from cardiac or pulmonary rehabilitation for those with prior exposure, and recognize that some subgroups may be differentially motivated to exercise. Our study also included patients before and after the start of the COVID-19 pandemic. Third, while we gathered substantial data on the use of individual components, our study design and nature of the intervention (“multicomponent package”) do not allow us to more definitively tease apart the effects of individual components. Fourth, we did not include eligibility criteria related to baseline physical activity levels. Future work should explore who benefits most from this intervention (eg, those with lower baseline physical activity levels) and whether baseline physical activity moderates one’s response to the intervention. Fifth, given that Mindful Steps did not focus on achieving a particular walking intensity, we did not collect reliable information on objective or subjective walking intensity throughout the intervention. Future research might examine the impact of Mindful Steps on other indices of physical activity, such as moderate-to-vigorous physical activity or sedentary time. Last, due to the COVID-19 pandemic, our trial procedures were necessarily changed midstudy to accommodate remote assessments and classes via videoconference. This change, however, provided valuable experience with remote study conduct in this population and strongly supported feasibility, increased access, and patient convenience (previously discussed in qualitative analysis) [57]. For example, participants emphasized the convenience of digital MBE classes, and also highlighted opportunities to foster more connection and discussion with peers and instructors [57].

Overall, the 12-month Mindful Steps intervention was feasible and acceptable, adherence was adequate, and a transition from in-person classes and testing visits to fully remote was feasible without significant challenges. We also found positive signals and sensitivity to change with outcomes of step counts, internal motivation, interoceptive awareness, and exercise self-efficacy. These findings suggest that a larger efficacy trial to examine the effects of Mindful Steps on promoting physical activity engagement and walking in patients with COPD and HF is warranted, and that fully remote conduct could be considered.

## Appendix

Multimedia Appendix 1 Difference in exploratory outcomes between Mindful Steps and usual care.

Multimedia Appendix 2 CONSORT-eHEALTH checklist (V 1.6.1).

## Abbreviations

CONSORT: Consolidated Standards of Reporting Trials
COPD: chronic obstructive pulmonary disease
ESC: Every Steps Count
HF: heart failure
HRQL: health-related quality of life
IMI: Intrinsic Motivation Inventory
MAIA: Multidimensional Assessment of Interoceptive Awareness
MBE: mind-body exercise
PROMIS: Patient-Reported Outcomes Measurement Information System
REDCap: Research Electronic Data Capture

## Data Availability

The datasets from this study are not publicly available due to concerns of participant confidentiality, but can be made available upon reasonable request to the principal investigator.
