# Supplementary material for: Promoting Walking in Cardiopulmonary Disease With Mindful Steps: Pilot Feasibility Randomized Controlled Trial of a Web-Based, Pedometer-Mediated Mind-Body Intervention
Source: JMIR Form Res. 2025 Oct 28;9:e74118. doi: 10.2196/74118 (PMC12605271; doi:10.2196/74118)
Supplement: Multimedia Appendix 1 [file formative_v9i1e74118_app1.docx]

Table S1. Difference in exploratory outcomes between Mindful Steps and usual care

|  | **Mindful Steps^a^** | | **Usual Care^b^** | |  |  |  |
| --- | --- | --- | --- | --- | --- | --- | --- |
|  | Mean (SD) | Difference from Baseline  Mean (SD) | Mean (SD) | Difference from Baseline  Mean (SD) | Difference in Differences (95% CI)^c,d^ | *P*-value^d^ | Cohen’s d of Difference in Differences |
| **SEES^e^** |  |  |  |  |  |  |  |
| Baseline | 6.38 (1.93) | 0 | 7.35 (2.24) | 0 | - | - |  |
| 3-month | 5.94 (2.25) | -.49 (1.97) | 6.08 (2.73) | -1.28 (2.32) | .80 (-.64, 2.23) | .274 | 0.38 |
| 6-month | 5.95 (2.42) | -.47 (1.89) | 5.55 (3.09) | -1.63 (2.67) | 1.27 (-.22, 2.76) | .093 | 0.53 |
| 9-month | 6.57 (2.48) | .11 (2.31) | 5.97 (3.22) | -1.20 (2.70) | 1.30 (-.22, 2.83) | .093 | 0.53 |
| 12-month | 7.01 (1.68) | .56 (1.29) | 6.02 (2.68) | -1.13 (2.23) | 1.77 (.20, 3.33) | .027 | 1.00 |
| **SEMCD^f^** |  |  |  |  |  |  |  |
| Baseline | 7.98 (1.71) | 0 | 7.76 (1.97) | 0 |  |  |  |
| 3-month | 7.53 (2.01) | -.43 (1.08) | 7.85 (2.17) | .14 (2.48) | -.57 (-1.66, .52) | .300 | -0.34 |
| 6-month | 7.79 (1.90) | -.38 (.78) | 6.71 (2.41) | -.83 (2.13) | .54 (-.58, 1.67) | .341 | 0.32 |
| 9-month | 8.46 (1.58) | .28 (1.26) | 7.15 (2.36) | -.39 (1.79) | .66 (-.49, 1.82) | .255 | 0.45 |
| 12-month | 7.82 (1.87) | -.36 (1.20) | 7.05 (2.40) | -.42 (2.28) | .15 (-1.03, 1.33) | .804 | 0.04 |
| **IMI^g^ Interest** |  |  |  |  |  |  |  |
| Baseline | 4.68 (1.77) | 0 | 4.33 (1.51) | 0 |  |  |  |
| 3-month | 4.63 (1.36) | -.06 (1.32) | 4.65 (1.83) | .07 (1.47) | .03 (-.98, 1.03) | .958 | -0.09 |
| 6-month | 5.39 (1.11) | .56 (1.46) | 4.18 (2.21) | -.40 (1.46) | 1.03 (.01, 2.04) | .047 | 0.66 |
| 9-month | 5.19 (1.48) | .43 (1.51) | 4.83 (1.71) | .25 (1.18) | .11 (-.92, 1.14) | .830 | 0.13 |
| 12-month | 4.95 (1.36) | .20 (1.69) | 4.51 (1.69) | -.25 (1.26) | .42 (-.64, 1.47) | .434 | 0.29 |
| **IMI^g^ Competence** |  |  |  |  |  |  |  |
| Baseline | 4.98 (1.50) | 0 | 4.21 (1.22) | 0 |  |  |  |
| 3-month | 5.19 (1.31) | .21 (1.17) | 4.42 (1.84) | -.12 (1.17) | .29 (-.63, 1.22) | .530 | 0.28 |
| 6-month | 5.70 (1.13) | .63 (1.62) | 4.12 (2.00) | -.27 (1.25) | .87 (-.07, 1.81) | .069 | 0.60 |
| 9-month | 5.65 (1.46) | .67 (1.70) | 4.95 (1.73) | .57 (1.09) | -.08 (-1.03, .88) | .875 | 0.07 |
| 12-month | 5.37 (1.63) | .39 (1.45) | 4.76 (1.44) | .42 (.77) | -.03 (-1.01, .95) | .949 | -0.02 |
| **IMI^g^ Choice** |  |  |  |  |  |  |  |
| Baseline | 4.86 (1.37) | 0 | 5.03 (1.11) | 0 |  |  |  |
| 3-month | 5.11 (1.36) | .28 (1.45) | 4.87 (1.48) | -.12 (1.87) | .40 (-.57, 1.37) | .414 | 0.25 |
| 6-month | 5.66 (.95) | .61 (1.14) | 5.00 (1.02) | .00 (1.54) | .81 (-.17, 1.79) | .104 | 0.47 |
| 9-month | 5.13 (1.23) | -.11 (.94) | 5.00 (1.24) | .00 (1.43) | .15 (-.86, 1.15) | .771 | -0.10 |
| 12-month | 5.62 (1.24) | .39 (1.33) | 4.96 (1.32) | -.09 (1.23) | .73 (-.30, 1.76) | .163 | 0.37 |
| **IMI^g^ Pressure** |  |  |  |  |  |  |  |
| Baseline | 2.34 (1.18) | 0 | 2.40 (1.22) | 0 |  |  |  |
| 3-month | 2.12 (.91) | -.27 (1.29) | 2.48 (1.65) | .08 (1.19) | -.38 (-1.33, .57) | .433 | -0.28 |
| 6-month | 1.90 (.75) | -.41 (1.20) | 3.52 (1.36) | 1.13 (1.01) | -1.59 (-2.55, -.63) | .002 | -1.36 |
| 9-month | 2.21 (.81) | -.13 (1.39) | 2.80 (1.45) | .42 (1.08) | -.49 (-1.48, .49) | .322 | -0.43 |
| 12-month | 2.28 (1.44) | -.05 (1.67) | 2.95 (1.47) | .67 (1.63) | -.75 (-1.76, .26) | .141 | -0.43 |
| **PAM^h^** |  |  |  |  |  |  |  |
| Baseline | 65.27 (10.43) | 0 | 71.39 (10.85) | 0 |  |  |  |
| 3-month | 67.18 (14.34) | 2.32 (9.21) | 67.98 (15.90) | -3.56 (12.48) | 5.88 (-3.53, 15.28) | .218 | 0.56 |
| 6-month | 67.97 (12.34) | 1.85 (13.56) | 74.52 (19.14) | 3.76 (13.97) | -1.66 (-11.50, 8.18) | .738 | -0.14 |
| 9-month | 74.71 (17.89) | 8.77 (13.65) | 79.47 (15.09) | 8.71 (11.38) | .23 (-9.82, 10.28) | .964 | 0.00 |
| 12-month | 74.02 (18.00) | 8.08 (20.66) | 78.71 (17.20) | 8.87 (10.73) | -.50 (-10.82, 9.82) | .923 | -0.04 |
| **MOS-SS Total^i^** |  |  |  |  |  |  |  |
| Baseline | 4.41 (.66) | 0 | 4.62 (.45) | 0 |  |  |  |
| 3-month | 4.39 (.65) | .01 (.49) | 4.34 (1.07) | -.26 (.65) | .27 (-.05, .59) | .095 | 0.49 |
| 6-month | 4.41 (.64) | .05 (.52) | 4.58 (.47) | .01 (.21) | .04 (-.29, .37) | .812 | 0.09 |
| 9-month | 4.44 (.75) | .05 (.35) | 4.72 (.48) | .15 (.35) | -.09 (-.43, .25) | .587 | -0.29 |
| 12-month | 4.48 (.72) | .09 (.42) | 4.48 (.85) | -.05 (.52) | .14 (-.20, .49) | .417 | 0.31 |
| **MAIA^j^ Body listen** |  |  |  |  |  |  |  |
| Baseline | 2.40 (1.41) | 0 | 2.38 (1.69) | 0 |  |  |  |
| 3-month | 2.93 (1.40) | .54 (1.40) | 1.92 (1.73) | -.62 (1.21) | 1.16 (.25, 2.07) | .013 | 0.87 |
| 6-month | 3.00 (1.32) | .48 (1.32) | 3.22 (1.51) | .75 (1.89) | -.13 (-1.08, .81) | .781 | -0.17 |
| 9-month | 3.16 (1.01) | .56 (1.22) | 3.03 (1.66) | .56 (1.20) | .10 (-.86, 1.07) | .830 | 0.00 |
| 12-month | 3.32 (1.26) | .72 (1.21) | 3.09 (1.57) | .70 (1.28) | .07 (-.92, 1.06) | .887 | 0.02 |
| **MAIA^j^ Emotional awareness** |  |  |  |  |  |  |  |
| Baseline | 3.35 (1.41) | 0 | 3.35 (1.21) | 0 |  |  |  |
| 3-month | 3.83 (1.25) | .45 (1.14) | 2.97 (1.67) | -.43 (.90) | .88 (.15, 1.61) | .019 | 0.83 |
| 6-month | 3.94 (1.05) | .36 (1.06) | 3.68 (1.39) | .42 (1.26) | .10 (-.65, .85) | .801 | -0.05 |
| 9-month | 3.82 (.91) | .22 (.95) | 3.23 (1.68) | -.03 (.91) | .43 (-.34, 1.19) | .274 | 0.27 |
| 12-month | 3.96 (.94) | .36 (.99) | 3.15 (1.50) | .02 (1.15) | .50 (-.29, 1.29) | .212 | 0.32 |
| **MAIA^j^ Not Distract** |  |  |  |  |  |  |  |
| Baseline | 2.01 (1.11) | 0 | 1.89 (1.12) | 0 |  |  |  |
| 3-month | 1.97 (.97) | .01 (1.12) | 2.69 (1.42) | .90 (1.36) | -.88 (-1.79, .02) | .056 | -0.74 |
| 6-month | 1.84 (1.15) | -.08 (1.35) | 1.97 (1.50) | .08 (1.63) | -.12 (-1.07, .82) | .798 | -0.11 |
| 9-month | 2.07 (1.25) | .12 (.89) | 2.69 (1.32) | .81 (1.41) | -.65 (-1.62, .31) | .183 | -0.62 |
| 12-month | 2.25 (1.05) | .30 (1.52) | 1.61 (1.44) | -.36 (1.46) | .69 (-.31, 1.68) | .173 | 0.44 |
| **MAIA^j^ Noticing** |  |  |  |  |  |  |  |
| Baseline | 3.48 (1.18) | 0 | 2.92 (1.60) | 0 |  |  |  |
| 3-month | 3.67 (1.22) | .13 (1.43) | 3.04 (1.61) | .29 (1.56) | -.16 (-1.20, .88) | .756 | -0.11 |
| 6-month | 3.64 (1.11) | .06 (1.35) | 2.73 (1.75) | .17 (2.21) | -.04 (-1.11, 1.03) | .943 | -0.06 |
| 9-month | 4.04 (.78) | .43 (1.22) | 3.23 (1.78) | .67 (2.01) | -.23 (-1.32, .86) | .682 | -0.15 |
| 12-month | 4.25 (.68) | .64 (1.28) | 3.48 (1.43) | .82 (1.85) | -.29 (-1.41, .83) | .610 | -0.12 |
| **MAIA^j^ Not Worry** |  |  |  |  |  |  |  |
| Baseline | 2.83 (1.24) | 0 | 3.44 (1.07) | 0 |  |  |  |
| 3-month | 3.08 (1.29) | .40 (1.19) | 3.67 (.88) | .23 (1.02) | .17 (-.57, .91) | .646 | 0.15 |
| 6-month | 2.97 (1.13) | .38 (.93) | 3.19 (.99) | -.22 (1.10) | .66 (-.11, 1.42) | .090 | 0.60 |
| 9-month | 2.91 (1.12) | .23 (.85) | 3.47 (1.12) | .06 (.91) | .20 (-.58, .99) | .607 | 0.19 |
| 12-month | 3.02 (1.07) | .33 (1.17) | 3.36 (1.19) | -.03 (1.28) | .39 (-.42, 1.19) | .342 | 0.30 |
| **MAIA^j^ Self-Regulation** |  |  |  |  |  |  |  |
| Baseline | 3.50 (.97) | 0 | 3.35 (1.32) | 0 |  |  |  |
| 3-month | 3.77 (.79) | .28 (.90) | 3.23 (1.66) | -.25 (1.75) | .53 (-.44, 1.50) | .280 | 0.42 |
| 6-month | 3.57 (1.01) | .04 (.92) | 3.79 (1.35) | .31 (2.31) | -.27 (-1.26, .72) | .588 | -0.17 |
| 9-month | 3.51 (.80) | .03 (.88) | 3.65 (1.47) | .17 (2.06) | -.17 (-1.18, .84) | .739 | -0.10 |
| 12-month | 3.87 (.91) | .38 (1.15) | 3.45 (1.48) | .02 (2.18) | .35 (-.68, 1.38) | .505 | 0.23 |
| **MAIA^j^ Trust** |  |  |  |  |  |  |  |
| Baseline | 3.92 (1.30) | 0 | 4.09 (1.21) | 0 |  |  |  |
| 3-month | 4.29 (.77) | .44 (1.33) | 4.49 (.75) | .10 (.79) | .34 (-.39, 1.07) | .356 | 0.29 |
| 6-month | 4.08 (.93) | .14 (1.17) | 3.94 (1.25) | -.39 (.60) | .63 (-.12, 1.38) | .096 | 0.53 |
| 9-month | 4.04 (1.18) | .21 (1.15) | 4.17 (.72) | -.17 (.80) | .36 (-.40, 1.13) | .348 | 0.37 |
| 12-month | 4.14 (1.04) | .32 (1.05) | 4.30 (.97) | -.06 (.51) | .35 (-.43, 1.14) | .371 | 0.42 |
| **MAIA^j^ Attention Regulation** |  |  |  |  |  |  |  |
| Baseline | 3.49 (1.01) | 0 | 3.70 (.99) | 0 |  |  |  |
| 3-month | 3.63 (.86) | .24 (.81) | 3.49 (1.47) | -.30 (1.38) | .53 (-.18, 1.25) | .138 | 0.52 |
| 6-month | 3.70 (.84) | .35 (1.00) | 3.35 (.95) | -.36 (1.37) | .72 (-.01, 1.45) | .054 | 0.62 |
| 9-month | 3.70 (.70) | .37 (.74) | 3.40 (1.04) | -.30 (1.40) | .65 (-.10, 1.40) | .090 | 0.64 |
| 12-month | 3.74 (.84) | .41 (.93) | 3.65 (.97) | -.14 (1.02) | .46 (-.31, 1.23) | .240 | 0.57 |
| **CES-D^k^** |  |  |  |  |  |  |  |
| Baseline | 8.62 (5.90) | 0 | 7.20 (5.39) | 0 |  |  |  |
| 3-month | 7.35 (6.58) | -1.87 (5.04) | 6.38 (6.25) | -1.15 (4.47) | -.56 (-5.04, 3.92) | .805 | -0.15 |
| 6-month | 8.19 (6.91) | -.76 (5.95) | 13.17 (12.19) | 5.42 (11.53) | -6.12 (-10.79, -1.46) | .011 | -0.74 |
| 9-month | 9.05 (8.30) | .58 (7.56) | 7.33 (5.33) | -.42 (5.20) | 1.16 (-3.60, 5.92) | .630 | 0.15 |
| 12-month | 5.83 (5.01) | -2.78 (4.17) | 7.36 (6.09) | 0 (7.32) | -2.45 (-7.38, 2.49) | .327 | -0.45 |
| **Cal/week moderate^l^** |  |  |  |  |  |  |  |
| Baseline | 6369.66 (4568.91) | 0 | 7482.19 (6634.28) | 0 |  |  |  |
| 3-month | 6806.51 (6639.96) | 32.22 (7476.08) | 7881.06 (6378.23) | -1070.78 (6257.13) | 1517.41 (-3442.90, 6477.72) | .544 | 0.16 |
| 6-month | 5698.22 (5561.77) | -299.25 (6343.20) | 10849.60 (9028.14) | 1678.69 (6339.66) | 138.21 (-4978.31, 5254.73) | .957 | -0.31 |
| 9-month | 6538.44 (5359.44) | 488.54 (6091.19) | 10493.44 (9291.68) | 1351.88 (11573.12) | -1111.70 (-6281.69, 4058.29) | .670 | -0.10 |
| 12-month | 7740.99 (5344.70) | 1916.94 (6086.87) | 8432.81 (5349.37) | -110.25 (4458.39) | 1940.07 (-3348.80, 7228.93) | .468 | 0.36 |
| **Freq/week moderate^m^** |  |  |  |  |  |  |  |
| Baseline | 2.42 (1.58) | 0 | 2.67 (2.23) | 0 |  |  |  |
| 3-month | 2.17 (1.49) | -.25 (2.01) | 2.64 (2.06) | 0 (1.52) | -.25 (-1.52, 1.02) | .696 | -0.14 |
| 6-month | 2.48 (1.94) | .14 (1.88) | 2.58 (2.02) | -.25 (1.86) | .40 (-.91, 1.72) | .546 | 0.21 |
| 9-month | 1.42 (1.30) | -.95 (1.22) | 2.58 (1.31) | -.25 (2.38) | -.81 (-2.17, .54) | .235 | -0.40 |
| 12-month | 1.89 (1.63) | -.47 (2.01) | 2.64 (1.69) | -.09 (1.87) | -.48 (-1.87, .91) | .494 | -0.19 |
| **SGRQ^n^** |  |  |  |  |  |  |  |
| Baseline | 37.42 (18.63) | 0 | 30.97 (12.12) | 0 |  |  |  |
| 3-month | 35.55 (16.51) | -2.11 (6.36) | 28.55 (15.15) | -2.42 (15.65) | .31 (-9.30, 9.92) | .949 | 0.03 |
| 6-month | 35.83 (15.01) | -1.16 (11.63) | 37.63 (18.64) | 7.06 (14.88) | -6.67 (-16.74, 3.39) | .190 | -0.64 |
| 9-month | 36.61 (18.74) | -.38 (10.53) | 32.16 (10.54) | 1.59 (7.57) | -1.20 (-11.37, 8.97) | .815 | -0.20 |
| 12-month | 35.02 (19.86) | -1.97 (13.06) | 34.40 (7.90) | 4.97 (12.69) | -7.08 (-17.63, 3.47) | .185 | -0.54 |
| **MLHFQ^0^** |  |  |  |  |  |  |  |
| Baseline | 2.82 (2.48) | 0 | .50 (.84) | 0 |  |  |  |
| 3-month | 2.55 (2.11) | -.50 (1.72) | 1.50 (1.87) | 1.20 (2.28) | -1.70 (-3.93, .54) | .129 | -0.89 |
| 6-month | 2.63 (1.85) | -.14 (2.19) | 1.50 (1.00) | .75 (.96) | -1.17 (-3.66, 1.32) | .342 | -0.48 |
| 9-month | 1.83 (2.14) | -.20 (1.64) | 1.00 (1.15) | .25 (1.26) | -.64 (-3.33, 2.04) | .624 | -0.30 |
| 12-month | 3.83 (3.19) | 1.80 (2.77) | 1.25 (1.26) | .50 (1.91) | 1.21 (-1.51, 3.93) | .368 | 0.53 |
| **PROMIS^p^ Pain** |  |  |  |  |  |  |  |
| Baseline | 49.64 (7.77) | 0 | 47.84 (7.59) | 0 |  |  |  |
| 3-month | 48.66 (9.11) | -.50 (7.06) | 50.16 (8.34) | 2.12 (4.23) | -2.62 (-8.03, 2.80) | .339 | -0.42 |
| 6-month | 48.73 (9.54) | .27 (10.00) | 48.08 (9.80) | .18 (7.27) | -.67 (-6.24, 4.90) | .812 | 0.01 |
| 9-month | 48.94 (11.12) | 1.24 (9.75) | 51.98 (9.51) | 4.09 (6.14) | -2.85 (-8.56, 2.85) | .323 | -0.33 |
| 12-month | 50.01 (7.53) | 2.31 (7.82) | 54.15 (12.43) | 5.60 (9.44) | -3.05 (-8.90, 2.80) | .303 | -0.39 |
| **PROMIS^p^ Fatigue** |  |  |  |  |  |  |  |
| Baseline | 48.50 (4.68) | 0 | 48.39 (7.13) | 0 |  |  |  |
| 3-month | 48.79 (6.17) | -.32 (5.87) | 48.07 (5.75) | -.81 (6.98) | .49 (-4.34, 5.32) | .840 | 0.08 |
| 6-month | 48.57 (7.00) | -.53 (6.83) | 49.82 (7.60) | .97 (7.66) | -1.85 (-6.87, 3.16) | .465 | -0.21 |
| 9-month | 47.25 (7.48) | -1.67 (7.43) | 49.83 (7.04) | .98 (6.07) | -2.33 (-7.51, 2.85) | .373 | -0.38 |
| 12-month | 47.36 (6.23) | -1.34 (6.31) | 48.19 (9.63) | .03 (9.63) | -.99 (-6.27, 4.29) | .709 | -0.18 |
| **PROMIS^p^ Physical Function** |  |  |  |  |  |  |  |
| Baseline | 43.62 (4.88) | 0 | 41.97 (4.79) | 0 |  |  |  |
| 3-month | 43.82 (6.19) | .55 (4.28) | 42.45 (6.46) | .22 (4.83) | .33 (-3.07, 3.73) | .849 | 0.07 |
| 6-month | 42.91 (4.91) | -.66 (4.70) | 41.93 (6.90) | -.42 (5.97) | -.02 (-3.53, 3.49) | .992 | -0.05 |
| 9-month | 43.50 (6.10) | .09 (4.67) | 43.94 (8.93) | 1.60 (7.09) | -1.96 (-5.55, 1.63) | .281 | -0.26 |
| 12-month | 42.76 (5.48) | -.65 (4.29) | 42.60 (6.59) | -.36 (5.64) | -.27 (3.95, 3.42) | .886 | -0.06 |

Note. ^a^N=26, 24, 21, 19, 19 at baseline, 3-month, 6-month, 9-month, 12-month, respectively; ^b^N=15, 13, 12, 12, 11 at baseline, 3-month, 6-month, 9-month, 12-month, respectively; ^c^Difference from baseline of Mindful Steps minus difference from baseline of Usual Care; ^d^Linear Mixed Effects Model testing the difference of differences from baseline between Mindful Steps and Usual Care; ^e^SEES: Self-Efficacy for Exercise Scale; ^f^SEMCD: Self-Efficacy for Managing Chronic Disease Scale; ^g^IMI: Intrinsic Motivation Inventory; ^h^PAM: Patient Activation Measure; ^i^MOS-SS: Medical Outcomes Study Social Support Survey; ^j^MAIA: Multidimensional Assessment of Interoceptive Awareness; ^k^CES-D: Center for Epidemiologic Studies Depression Scale; ^l^Cal week moderate: Calories of moderate intensity exercise per week, calculated from the Community Healthy Activities Model Program for Seniors Physical Activity Questionnaire; ^m^Freq/week moderate: Frequency of moderate intensity exercise per week, calculated from the Community Healthy; Activities Model Program for Seniors Physical Activity Questionnaire; ^n^SGRQ: St. George’s Respiratory Questionnaire; ^o^MLHFQ: Minnesota Living with Heart Failure Questionnaire; ^p^PROMIS: Patient-Reported Outcomes Measurement Information System
