# Supplementary material for: Promoting Walking in Cardiopulmonary Disease With Mindful Steps: Pilot Feasibility Randomized Controlled Trial of a Web-Based, Pedometer-Mediated Mind-Body Intervention
Source: JMIR Form Res. 2025 Oct 28;9:e74118. doi: 10.2196/74118 (PMC12605271; doi:10.2196/74118)
Supplement: Multimedia Appendix 2 [file formative_v9i1e74118_app2.pdf]

CONSORT-EHEALTH (V 1.6.1) - Submission/Publication Form

The CONSORT-EHEALTH checklist is intended for authors of randomized trials evaluating web-based and Internet-based applications/interventions, including mobile interventions, electronic games (incl multiplayer games), social media, certain telehealth applications, and other interactive and/or networked electronic applications. Some of the items (e.g. all subitems under item 5 - description of the intervention) may also be applicable for other study designs.

The goal of the CONSORT EHEALTH checklist and guideline is to be
a) a guide for reporting for authors of RCTs,
b) to form a basis for appraisal of an ehealth trial (in terms of validity)

CONSORT-EHEALTH items/subitems are MANDATORY reporting items for studies published in the Journal of Medical Internet Research and other journals / scientific societies endorsing the checklist.

Items numbered 1., 2., 3., 4a., 4b etc are original CONSORT or CONSORT-NPT (non-pharmacologic treatment) items.
Items with Roman numerals (i., ii, iii, iv etc.) are CONSORT-EHEALTH extensions/clarifications.

As the CONSORT-EHEALTH checklist is still considered in a formative stage, we would ask that you also RATE ON A SCALE OF 1-5 how important/useful you feel each item is FOR THE PURPOSE OF THE CHECKLIST and reporting guideline (optional).

Mandatory reporting items are marked with a red *.
In the textboxes, either copy & paste the relevant sections from your manuscript into this form - please include any quotes from your manuscript in QUOTATION MARKS,
or answer directly by providing additional information not in the manuscript, or elaborating on why the item was not relevant for this study.

YOUR ANSWERS WILL BE PUBLISHED AS A SUPPLEMENTARY FILE TO YOUR PUBLICATION IN JMIR AND ARE CONSIDERED PART OF YOUR PUBLICATION (IF ACCEPTED).
Please fill in these questions diligently. Information will not be copyedited, so please use proper spelling and grammar, use correct capitalization, and avoid abbreviations.

DO NOT FORGET TO SAVE AS PDF _AND_ CLICK THE SUBMIT BUTTON SO YOUR ANSWERS ARE IN OUR DATABASE !!!

Citation Suggestion (if you append the pdf as Appendix we suggest to cite this paper in the caption):
Eysenbach G, CONSORT-EHEALTH Group

CONSORT-EHEALTH: Improving and Standardizing Evaluation Reports of Web-based and Mobile Health Interventions
J Med Internet Res 2011;13(4):e126
URL: http://www.jmir.org/2011/4/e126/
doi: 10.2196/jmir.1923
PMID: 22209829

Sign in to Google to save your progress. Learn more

* Indicates required question

Your name *

First Last

Kristen Kraemer

Primary Affiliation (short), City, Country *

University of Toronto, Toronto, Canada

Beth Israel Deaconess Medical Center

Your e-mail address *

abc@gmail.com

kkraemer@bidmc.harvard.edu

Title of your manuscript *

Provide the (draft) title of your manuscript.

Promoting Walking in Cardiopulmonary Disease With Mindful Steps: A Pilot Feasibility Randomized Controlled Trial Study of a Web-Based, Pedometer-Mediated Mind-Body Intervention

Name of your App/Software/Intervention *

If there is a short and a long/alternate name, write the short name first and add the long name in brackets.

Mindful Steps

Evaluated Version (if any)

e.g. "V1", "Release 2017-03-01", "Version 2.0.27913"

Your answer

Language(s) *

What language is the intervention/app in? If multiple languages are available, separate by comma (e.g. "English, French")

English

URL of your Intervention Website or App

e.g. a direct link to the mobile app on app in appstore (itunes, Google Play), or URL of the website. If the intervention is a DVD or hardware, you can also link to an Amazon page.

Your answer

URL of an image/screenshot (optional)

Your answer

Accessibility *

Can an enduser access the intervention presently?

- ○ access is free and open
- ◉ access only for special usergroups, not open
- ○ access is open to everyone, but requires payment/subscription/in-app purchases
- ○ app/intervention no longer accessible
- ○ Other:

Primary Medical Indication/Disease/Condition *

e.g. "Stress", "Diabetes", or define the target group in brackets after the condition, e.g. "Autism (Parents of children with)", "Alzheimers (Informal Caregivers of)"

Chronic Obstructive Pulmonary Disease or hea

Primary Outcomes measured in trial *

comma-separated list of primary outcomes reported in the trial

feasibility, acceptability

Secondary/other outcomes

Are there any other outcomes the intervention is expected to affect?

Your answer

Recommended "Dose" *

What do the instructions for users say on how often the app should be used?

- (•) Approximately Daily
- ( ) Approximately Weekly
- ( ) Approximately Monthly
- ( ) Approximately Yearly
- ( ) "as needed"
- ( ) Other:

Approx. Percentage of Users (starters) still using the app as recommended after 3 months *

- (•) unknown / not evaluated
- ( ) 0-10%
- ( ) 11-20%
- ( ) 21-30%
- ( ) 31-40%
- ( ) 41-50%
- ( ) 51-60%
- ( ) 61-70%
- ( ) 71%-80%
- ( ) 81-90%
- ( ) 91-100%
- ( ) Other:

Overall, was the app/intervention effective? *

- ( ) yes: all primary outcomes were significantly better in intervention group vs control
- ( ) partly: SOME primary outcomes were significantly better in intervention group vs control
- ( ) no statistically significant difference between control and intervention
- ( ) potentially harmful: control was significantly better than intervention in one or more outcomes
- ( ) inconclusive: more research is needed
- (x) Other: This trial did not assess efficacy - feasibility trial

Article Preparation Status/Stage *

At which stage in your article preparation are you currently (at the time you fill in this form)

- ( ) not submitted yet - in early draft status
- ( ) not submitted yet - in late draft status, just before submission
- ( ) submitted to a journal but not reviewed yet
- ( ) submitted to a journal and after receiving initial reviewer comments
- (x) submitted to a journal and accepted, but not published yet
- ( ) published
- ( ) Other:

Journal *

If you already know where you will submit this paper (or if it is already submitted), please provide the journal name (if it is not JMIR, provide the journal name under "other")

- ○ not submitted yet / unclear where I will submit this
- ○ Journal of Medical Internet Research (JMIR)
- ○ JMIR mHealth and UHealth
- ○ JMIR Serious Games
- ○ JMIR Mental Health
- ○ JMIR Public Health
- ◉ JMIR Formative Research
- ○ Other JMIR sister journal
- ○ Other:

Is this a full powered effectiveness trial or a pilot/feasibility trial? *

- ◉ Pilot/feasibility
- ○ Fully powered

Manuscript tracking number *

If this is a JMIR submission, please provide the manuscript tracking number under "other" (The ms tracking number can be found in the submission acknowledgement email, or when you login as author in JMIR. If the paper is already published in JMIR, then the ms tracking number is the four-digit number at the end of the DOI, to be found at the bottom of each published article in JMIR)

- ○ no ms number (yet) / not (yet) submitted to / published in JMIR
- ◉ Other: 74118

TITLE AND ABSTRACT

1a) TITLE: Identification as a randomized trial in the title

1a) Does your paper address CONSORT item 1a? *

I.e does the title contain the phrase "Randomized Controlled Trial"? (if not, explain the reason under "other")

- (selected) yes
- Other:

1a-i) Identify the mode of delivery in the title

Identify the mode of delivery. Preferably use “web-based” and/or “mobile” and/or “electronic game” in the title. Avoid ambiguous terms like “online”, “virtual”, “interactive”. Use “Internet-based” only if Intervention includes non-web-based Internet components (e.g. email), use “computer-based” or “electronic” only if offline products are used. Use “virtual” only in the context of “virtual reality” (3-D worlds). Use “online” only in the context of “online support groups”. Complement or substitute product names with broader terms for the class of products (such as “mobile” or “smart phone” instead of “iphone”), especially if the application runs on different platforms.

	1	2	3	4	5	
subitem not at all important	○	○	○	○	○	essential

Does your paper address subitem 1a-i? *

Copy and paste relevant sections from manuscript title (include quotes in quotation marks "like this" to indicate direct quotes from your manuscript), or elaborate on this item by providing additional information not in the ms, or briefly explain why the item is not applicable/relevant for your study

Promoting Walking in Cardiopulmonary Disease With Mindful Steps: A Pilot Feasibility Randomized Controlled Trial Study of a Web-Based, Pedometer-Mediated Mind-Body Intervention

1a-ii) Non-web-based components or important co-interventions in title

Mention non-web-based components or important co-interventions in title, if any (e.g., "with telephone support").

	1	2	3	4	5	
subitem not at all important	○	○	○	○	○	essential

Does your paper address subitem 1a-ii?

Copy and paste relevant sections from manuscript title (include quotes in quotation marks "like this" to indicate direct quotes from your manuscript), or elaborate on this item by providing additional information not in the ms, or briefly explain why the item is not applicable/relevant for your study

Your answer

1a-iii) Primary condition or target group in the title

Mention primary condition or target group in the title, if any (e.g., “for children with Type I Diabetes”) Example: A Web-based and Mobile Intervention with Telephone Support for Children with Type I Diabetes: Randomized Controlled Trial

	1	2	3	4	5	
subitem not at all important	○	○	○	○	○	essential

Does your paper address subitem 1a-iii? *

Copy and paste relevant sections from manuscript title (include quotes in quotation marks "like this" to indicate direct quotes from your manuscript), or elaborate on this item by providing additional information not in the ms, or briefly explain why the item is not applicable/relevant for your study

"Cardiopulmonary Disease"

1b) ABSTRACT: Structured summary of trial design, methods, results, and conclusions

NPT extension: Description of experimental treatment, comparator, care providers, centers, and blinding status.

1b-i) Key features/functionalities/components of the intervention and comparator in the METHODS section of the ABSTRACT

Mention key features/functionalities/components of the intervention and comparator in the abstract. If possible, also mention theories and principles used for designing the site. Keep in mind the needs of systematic reviewers and indexers by including important synonyms. (Note: Only report in the abstract what the main paper is reporting. If this information is missing from the main body of text, consider adding it)

	1	2	3	4	5	
subitem not at all important	○	○	○	○	○	essential

Does your paper address subitem 1b-i? *

Copy and paste relevant sections from the manuscript abstract (include quotes in quotation marks "like this" to indicate direct quotes from your manuscript), or elaborate on this item by providing additional information not in the ms, or briefly explain why the item is not applicable/relevant for your study

"pilot randomized controlled trial"; "randomized to Mindful Steps or usual care in a 2:1 ratio"

1b-ii) Level of human involvement in the METHODS section of the ABSTRACT

Clarify the level of human involvement in the abstract, e.g., use phrases like “fully automated” vs. “therapist/nurse/care provider/physician-assisted” (mention number and expertise of providers involved, if any). (Note: Only report in the abstract what the main paper is reporting. If this information is missing from the main body of text, consider adding it)

	1	2	3	4	5	
subitem not at all important	○	○	○	○	○	essential

Does your paper address subitem 1b-ii?

Copy and paste relevant sections from the manuscript abstract (include quotes in quotation marks "like this" to indicate direct quotes from your manuscript), or elaborate on this item by providing additional information not in the ms, or briefly explain why the item is not applicable/relevant for your study

this was not addressed in the abstract

1b-iii) Open vs. closed, web-based (self-assessment) vs. face-to-face assessments in the METHODS section of the ABSTRACT

Mention how participants were recruited (online vs. offline), e.g., from an open access website or from a clinic or a closed online user group (closed usergroup trial), and clarify if this was a purely web-based trial, or there were face-to-face components (as part of the intervention or for assessment). Clearly say if outcomes were self-assessed through questionnaires (as common in web-based trials). Note: In traditional offline trials, an open trial (open-label trial) is a type of clinical trial in which both the researchers and participants know which treatment is being administered. To avoid confusion, use “blinded” or “unblinded” to indicated the level of blinding instead of “open”, as “open” in web-based trials usually refers to “open access” (i.e. participants can self-enrol). (Note: Only report in the abstract what the main paper is reporting. If this information is missing from the main body of text, consider adding it)

	1	2	3	4	5	
subitem not at all important	○	○	○	○	○	essential

Does your paper address subitem 1b-iii?

Copy and paste relevant sections from the manuscript abstract (include quotes in quotation marks "like this" to indicate direct quotes from your manuscript), or elaborate on this item by providing additional information not in the ms, or briefly explain why the item is not applicable/relevant for your study

"Feasibility (recruitment rate, retention), intervention acceptability, and intervention adherence were the primary outcomes. Exploratory outcomes assessed at baseline, 3-, 6-, 9-, and 12-months included daily step counts, cognitive-behavioral/psychosocial measures, health-related quality of life, and self-reported physical function"

1b-iv) RESULTS section in abstract must contain use data

Report number of participants enrolled/assessed in each group, the use/uptake of the intervention (e.g., attrition/adherence metrics, use over time, number of logins etc.), in addition to primary/secondary outcomes. (Note: Only report in the abstract what the main paper is reporting. If this information is missing from the main body of text, consider adding it)

	1	2	3	4	5	
subitem not at all important	○	○	○	○	○	essential

Does your paper address subitem 1b-iv?

Copy and paste relevant sections from the manuscript abstract (include quotes in quotation marks "like this" to indicate direct quotes from your manuscript), or elaborate on this item by providing additional information not in the ms, or briefly explain why the item is not applicable/relevant for your study

"Forty-one participants were randomized to Mindful Steps (n=26) or usual care (n=15)." "In the intervention group, over 12 months, 58% (15/26) met a pre-defined benchmark for mind-body exercise class adherence (attending >70% of classes)" "There was a positive signal regarding group differences in the change in daily steps counts from baseline, favoring intervention at 3-months (estimate = 2038.77 steps per day between groups, 95% CI, 289.76-3788.77), 6-months (estimate = 3031.45, 95% CI, 1261.15-4801.74), and 9-months (estimate = 2703.80, 95% CI, 862.97-4544.62)."

1b-v) CONCLUSIONS/DISCUSSION in abstract for negative trials

Conclusions/Discussions in abstract for negative trials: Discuss the primary outcome - if the trial is negative (primary outcome not changed), and the intervention was not used, discuss whether negative results are attributable to lack of uptake and discuss reasons. (Note: Only report in the abstract what the main paper is reporting. If this information is missing from the main body of text, consider adding it)

	1	2	3	4	5	
subitem not at all important	○	○	○	○	○	essential

Does your paper address subitem 1b-v?

Copy and paste relevant sections from the manuscript abstract (include quotes in quotation marks "like this" to indicate direct quotes from your manuscript), or elaborate on this item by providing additional information not in the ms, or briefly explain why the item is not applicable/relevant for your study

"Mindful Steps was largely feasible, acceptable, and had adequate intervention engagement. There were positive signals favoring the multimodal web intervention for daily step counts, interoceptive awareness, internal awareness, and exercise self-efficacy that will inform hypotheses in future studies"

INTRODUCTION

2a) In INTRODUCTION: Scientific background and explanation of rationale

2a-i) Problem and the type of system/solution

Describe the problem and the type of system/solution that is object of the study: intended as stand-alone intervention vs. incorporated in broader health care program? Intended for a particular patient population? Goals of the intervention, e.g., being more cost-effective to other interventions, replace or complement other solutions? (Note: Details about the intervention are provided in “Methods” under 5)

	1	2	3	4	5	
subitem not at all important	○	○	○	○	○	essential

Does your paper address subitem 2a-i? *

Copy and paste relevant sections from the manuscript (include quotes in quotation marks "like this" to indicate direct quotes from your manuscript), or elaborate on this item by providing additional information not in the ms, or briefly explain why the item is not applicable/relevant for your study

"Mindful Steps was thus designed to incorporate mind-body training as an adaptation of this original web-based intervention to further bolster and better sustain effects on walking"

2a-ii) Scientific background, rationale: What is known about the (type of) system

Scientific background, rationale: What is known about the (type of) system that is the object of the study (be sure to discuss the use of similar systems for other conditions/diagnoses, if appropiate), motivation for the study, i.e. what are the reasons for and what is the context for this specific study, from which stakeholder viewpoint is the study performed, potential impact of findings [2]. Briefly justify the choice of the comparator.

	1	2	3	4	5	
subitem not at all important	○	○	○	○	○	essential

Does your paper address subitem 2a-ii? *

Copy and paste relevant sections from the manuscript (include quotes in quotation marks "like this" to indicate direct quotes from your manuscript), or elaborate on this item by providing additional information not in the ms, or briefly explain why the item is not applicable/relevant for your study

"Moy and et al [30,31] developed an accessible, web-based, pedometer-mediated intervention to promote walking behaviors among individuals with COPD called every step counts (ESC) [30-37]. The 6-month intervention, based on self-regulation theories of health behavior change [38], included a web platform coupled with a pedometer, which provided individualized step-count goals, iterative feedback graphically and on the face of the pedometer, educational tips for disease self-management, motivational messages to help sustain walking, and an online community forum for social support. The intervention was safe, engaging, and associated with improvements in daily step counts and HRQL at four 4 or six 6 months compared to with controls [30-33]. However, daily step count and HRQL improvements were not maintained at the 12-month follow-up [32]. Mindful Steps was thus designed to incorporate mind-body training as an adaptation of this original web-based intervention to further bolster and better sustain effects on walking."

2b) In INTRODUCTION: Specific objectives or hypotheses

Does your paper address CONSORT subitem 2b? *

Copy and paste relevant sections from the manuscript (include quotes in quotation marks "like this" to indicate direct quotes from your manuscript), or elaborate on this item by providing additional information not in the ms, or briefly explain why the item is not applicable/relevant for your study

"The aim of this pilot randomized controlled trial was to examine the feasibility and acceptability of Mindful Steps among individuals with COPD and HF and explore preliminary effect estimates on physical activity objectively measured as daily step counts, disease-specific HRQL, cognitive and psychosocial outcomes, and self-reported physical function compared with usual care"

METHODS

3a) Description of trial design (such as parallel, factorial) including allocation ratio

Does your paper address CONSORT subitem 3a? *

Copy and paste relevant sections from the manuscript (include quotes in quotation marks "like this" to indicate direct quotes from your manuscript), or elaborate on this item by providing additional information not in the ms, or briefly explain why the item is not applicable/relevant for your study

"Individuals with COPD and/or HF were randomized in a 2:1 ratio to a 12-month Mindful Steps intervention or usual care."

3b) Important changes to methods after trial commencement (such as eligibility criteria), with reasons

Does your paper address CONSORT subitem 3b? *

Copy and paste relevant sections from the manuscript (include quotes in quotation marks "like this" to indicate direct quotes from your manuscript), or elaborate on this item by providing additional information not in the ms, or briefly explain why the item is not applicable/relevant for your study

N/A no changes to the methods

3b-i) Bug fixes, Downtimes, Content Changes

Bug fixes, Downtimes, Content Changes: ehealth systems are often dynamic systems. A description of changes to methods therefore also includes important changes made on the intervention or comparator during the trial (e.g., major bug fixes or changes in the functionality or content) (5-iii) and other “unexpected events” that may have influenced study design such as staff changes, system failures/downtimes, etc. [2].

	1	2	3	4	5	
subitem not at all important	○	○	○	○	○	essential

Does your paper address subitem 3b-i?

Copy and paste relevant sections from the manuscript (include quotes in quotation marks "like this" to indicate direct quotes from your manuscript), or elaborate on this item by providing additional information not in the ms, or briefly explain why the item is not applicable/relevant for your study

"In-person MBE classes were transitioned to live videoconferencing via Starleaf (the hospital's preferred meeting platform in 2020) during the COVID-19 pandemic in March 2020."

4a) Eligibility criteria for participants

Does your paper address CONSORT subitem 4a? *

Copy and paste relevant sections from the manuscript (include quotes in quotation marks "like this" to indicate direct quotes from your manuscript), or elaborate on this item by providing additional information not in the ms, or briefly explain why the item is not applicable/relevant for your study

"Individuals were eligible if they met the following criteria: (1) age over older than >40 years; (2) clinical diagnosis of COPD, defined as either a ratio of forced expiratory volume in one second (FEV1) to forced vital capacity <0.70 or chest computed tomography evidence of emphysema, and/or clinical diagnosis of HF syndrome (with left ventricular systolic dysfunction or preserved ejection fraction, and New York Heart Association Class 1-3); (3) medical clearance from a provider to participate in an exercise program; (4) an active email account with the ability to check email at least weekly; and (5) access to a computer with an internet connection, USB port, and Windows. Individuals were excluded if they met any of the following criteria: (1) self-reported COPD or HF exacerbation in the previous 2 weeks; (2) inability to ambulate; (3) clinical signs of unstable cardiovascular disease; (4) hypoxemia during 6-minute walk test (oxygen saturation <85% with supplemental oxygen); (5) inability to collect at least 7 of 14 days of baseline step counts; and (6) current participation in a cardiac or pulmonary rehabilitation program."

4a-i) Computer / Internet literacy

Computer / Internet literacy is often an implicit “de facto” eligibility criterion - this should be explicitly clarified.

	1	2	3	4	5	
subitem not at all important	○	○	○	○	○	essential

Does your paper address subitem 4a-i?

Copy and paste relevant sections from the manuscript (include quotes in quotation marks "like this" to indicate direct quotes from your manuscript), or elaborate on this item by providing additional information not in the ms, or briefly explain why the item is not applicable/relevant for your study

Eligibility included "access to a computer with an internet connection, USB port, and Windows"

4a-ii) Open vs. closed, web-based vs. face-to-face assessments:

Open vs. closed, web-based vs. face-to-face assessments: Mention how participants were recruited (online vs. offline), e.g., from an open access website or from a clinic, and clarify if this was a purely web-based trial, or there were face-to-face components (as part of the intervention or for assessment), i.e., to what degree got the study team to know the participant. In online-only trials, clarify if participants were quasi-anonymous and whether having multiple identities was possible or whether technical or logistical measures (e.g., cookies, email confirmation, phone calls) were used to detect/prevent these.

	1	2	3	4	5	
subitem not at all important	○	○	○	○	○	essential

Does your paper address subitem 4a-ii? *

Copy and paste relevant sections from the manuscript (include quotes in quotation marks "like this" to indicate direct quotes from your manuscript), or elaborate on this item by providing additional information not in the ms, or briefly explain why the item is not applicable/relevant for your study

"Potential participants, identified through hospital registries, physician clinic schedules (cardiology and pulmonology), and specialist and primary care physician referrals, were sent an opt-out mailing and contacted by telephone by research assistants from February 2019 to May 2020. "

4a-iii) Information giving during recruitment

Information given during recruitment. Specify how participants were briefed for recruitment and in the informed consent procedures (e.g., publish the informed consent documentation as appendix, see also item X26), as this information may have an effect on user self-selection, user expectation and may also bias results.

	1	2	3	4	5	
subitem not at all important	○	○	○	○	○	essential

Does your paper address subitem 4a-iii?

Copy and paste relevant sections from the manuscript (include quotes in quotation marks "like this" to indicate direct quotes from your manuscript), or elaborate on this item by providing additional information not in the ms, or briefly explain why the item is not applicable/relevant for your study

"Potential participants, identified through hospital registries, physician clinic schedules (cardiology and pulmonology), and specialist and primary care physician referrals, were sent an opt-out mailing and contacted by telephone by research assistants from February 2019 to May 2020. Participants were also recruited via flyers, digital screen ads in specialist and primary care clinic waiting rooms, and in-person presentations at local cardiac or pulmonary rehabilitation programs (for those who completed or no longer engaged in the program). Interested individuals completed a phone screen and scheduled an in-person consent visit."

4b) Settings and locations where the data were collected

Does your paper address CONSORT subitem 4b? *

Copy and paste relevant sections from the manuscript (include quotes in quotation marks "like this" to indicate direct quotes from your manuscript), or elaborate on this item by providing additional information not in the ms, or briefly explain why the item is not applicable/relevant for your study

"Participants completed a 14-day run-in period where they were asked to wear the pedometer (Fitbit Alta HR; Fitbit Inc, San Francisco, CA, USA) all waking hours. Seven days of valid data (>200 steps per day) were required for randomization. Participants were then randomized in a 2:1 ratio to Mindful Steps or usual care using block randomization with varying/unpredictable block sizes. The random numbers were computer-generated and concealed by sealed and numbered opaque envelopes. After eligibility was confirmed, research assistants opened the next envelope in sequence and informed the participant of their assignment. The study was conducted in four 4 waves (cohorts of ~≈10 participants each). During the COVID-19 pandemic, all study activities, including recruitment, consent, and testing, were transitioned to all-remote procedures in March 2020."

4b-i) Report if outcomes were (self-)assessed through online questionnaires

Clearly report if outcomes were (self-)assessed through online questionnaires (as common in web-based trials) or otherwise.

	1	2	3	4	5	
subitem not at all important	○	○	○	○	○	essential

Does your paper address subitem 4b-i? *

Copy and paste relevant sections from the manuscript (include quotes in quotation marks "like this" to indicate direct quotes from your manuscript), or elaborate on this item by providing additional information not in the ms, or briefly explain why the item is not applicable/relevant for your study

"Study data were stored confidentially using Research Electronic Data Capture (REDCap), which is Health Insurance Portability and Accountability Act–compliant, password-protected, and managed by Beth Israel Deaconess Medical Center"

4b-ii) Report how institutional affiliations are displayed

Report how institutional affiliations are displayed to potential participants [on ehealth media], as affiliations with prestigious hospitals or universities may affect volunteer rates, use, and reactions with regards to an intervention.(Not a required item – describe only if this may bias results)

	1	2	3	4	5	
subitem not at all important	○	○	○	○	○	essential

Does your paper address subitem 4b-ii?

Copy and paste relevant sections from the manuscript (include quotes in quotation marks "like this" to indicate direct quotes from your manuscript), or elaborate on this item by providing additional information not in the ms, or briefly explain why the item is not applicable/relevant for your study

Your answer

5) The interventions for each group with sufficient details to allow replication, including how and when they were actually administered

5-i) Mention names, credential, affiliations of the developers, sponsors, and owners

Mention names, credential, affiliations of the developers, sponsors, and owners [6] (if authors/evaluators are owners or developer of the software, this needs to be declared in a “Conflict of interest” section or mentioned elsewhere in the manuscript).

	1	2	3	4	5	
subitem not at all important	○	○	○	○	○	essential

Does your paper address subitem 5-i?

Copy and paste relevant sections from the manuscript (include quotes in quotation marks "like this" to indicate direct quotes from your manuscript), or elaborate on this item by providing additional information not in the ms, or briefly explain why the item is not applicable/relevant for your study

"Moy and et al [30,31] developed an accessible, web-based, pedometer-mediated intervention to promote walking behaviors among individuals with COPD called every step counts (ESC) "

5-ii) Describe the history/development process

Describe the history/development process of the application and previous formative evaluations (e.g., focus groups, usability testing), as these will have an impact on adoption/use rates and help with interpreting results.

	1	2	3	4	5	
subitem not at all important	○	○	○	○	○	essential

Does your paper address subitem 5-ii?

Copy and paste relevant sections from the manuscript (include quotes in quotation marks "like this" to indicate direct quotes from your manuscript), or elaborate on this item by providing additional information not in the ms, or briefly explain why the item is not applicable/relevant for your study

"Moy and et al [30,31] developed an accessible, web-based, pedometer-mediated intervention to promote walking behaviors among individuals with COPD called every step counts (ESC) [30-37]. The 6-month intervention, based on self-regulation theories of health behavior change [38], included a web platform coupled with a pedometer, which provided individualized step-count goals, iterative feedback graphically and on the face of the pedometer, educational tips for disease self-management, motivational messages to help sustain walking, and an online community forum for social support. The intervention was safe, engaging, and associated with improvements in daily step counts and HRQL at four 4 or six 6 months compared to with controls [30-33]. However, daily step count and HRQL improvements were not maintained at the 12-month follow-up [32]. Mindful Steps was thus designed to incorporate mind-body training as an adaptation of this original web-based intervention to further bolster and better sustain effects on walking."

5-iii) Revisions and updating

Revisions and updating. Clearly mention the date and/or version number of the application/intervention (and comparator, if applicable) evaluated, or describe whether the intervention underwent major changes during the evaluation process, or whether the development and/or content was “frozen” during the trial. Describe dynamic components such as news feeds or changing content which may have an impact on the replicability of the intervention (for unexpected events see item 3b).

	1	2	3	4	5	
subitem not at all important	○	○	○	○	○	essential

Does your paper address subitem 5-iii?

Copy and paste relevant sections from the manuscript (include quotes in quotation marks "like this" to indicate direct quotes from your manuscript), or elaborate on this item by providing additional information not in the ms, or briefly explain why the item is not applicable/relevant for your study

n/a did not undergo major changes during evaluation process

5-iv) Quality assurance methods

Provide information on quality assurance methods to ensure accuracy and quality of information provided [1], if applicable.

	1	2	3	4	5	
subitem not at all important	○	○	○	○	○	essential

Does your paper address subitem 5-iv?

Copy and paste relevant sections from the manuscript (include quotes in quotation marks "like this" to indicate direct quotes from your manuscript), or elaborate on this item by providing additional information not in the ms, or briefly explain why the item is not applicable/relevant for your study

n/a this was not included in the mansucript

5-v) Ensure replicability by publishing the source code, and/or providing screenshots/screen-capture video, and/or providing flowcharts of the algorithms used

Ensure replicability by publishing the source code, and/or providing screenshots/screen-capture video, and/or providing flowcharts of the algorithms used. Replicability (i.e., other researchers should in principle be able to replicate the study) is a hallmark of scientific reporting.

	1	2	3	4	5	
subitem not at all important	○	○	○	○	○	essential

Does your paper address subitem 5-v?

Copy and paste relevant sections from the manuscript (include quotes in quotation marks "like this" to indicate direct quotes from your manuscript), or elaborate on this item by providing additional information not in the ms, or briefly explain why the item is not applicable/relevant for your study

n/a given that this is a feasibility study

5-vi) Digital preservation

Digital preservation: Provide the URL of the application, but as the intervention is likely to change or disappear over the course of the years; also make sure the intervention is archived (Internet Archive, webcitation.org, and/or publishing the source code or screenshots/videos alongside the article). As pages behind login screens cannot be archived, consider creating demo pages which are accessible without login.

	1	2	3	4	5	
subitem not at all important	○	○	○	○	○	essential

Does your paper address subitem 5-vi?

Copy and paste relevant sections from the manuscript (include quotes in quotation marks "like this" to indicate direct quotes from your manuscript), or elaborate on this item by providing additional information not in the ms, or briefly explain why the item is not applicable/relevant for your study

n/a this was not included in the manuscript

5-vii) Access

Access: Describe how participants accessed the application, in what setting/context, if they had to pay (or were paid) or not, whether they had to be a member of specific group. If known, describe how participants obtained “access to the platform and Internet” [1]. To ensure access for editors/reviewers/readers, consider to provide a “backdoor” login account or demo mode for reviewers/readers to explore the application (also important for archiving purposes, see vi).

	1	2	3	4	5	
subitem not at all important	○	○	○	○	○	essential

Does your paper address subitem 5-vii? *

Copy and paste relevant sections from the manuscript (include quotes in quotation marks "like this" to indicate direct quotes from your manuscript), or elaborate on this item by providing additional information not in the ms, or briefly explain why the item is not applicable/relevant for your study

"This was a 12-month multicomponent web-based, pedometer-mediated intervention that included: (1) a pedometer (Fitbit Alta HR), (2) the Mindful Steps web platform with mind-body videos, motivational messages, educational content, online community forum, and (3) live mind-body exercise (MBE) classes.
The pedometer is paired with the Mindful Steps web platform to visually display the participant's daily/weekly steps and individualized step goals. Participants were asked to upload their step counts to the study server at least once a week. The initial step-count goal was calculated using an algorithm designed to promote slow and safe increases, where the most recent seven 7 consecutive days of synced data were averaged and 400 steps were added. Previous research among individuals with type 2 diabetes used an 800-step increment based on participant feedback [52]. Subsequent research selected a 400-step increment in COPD as a more attainable goal in this population [31]. Subsequent goals could increase, decrease, or stay the same, but not go up by more than 400 steps over the previous goal and not exceed 10,000 steps for safety reasons. In the absence of new data, the prior week's goal was re-issued. Participants were asked to wear the wrist-worn pedometer daily during waking hours. Motivational messages and educational tips focused on self-managing HF and COPD and walking-related topics (ege.g., "Managing Stress and Anxiety," "Handling Setbacks," and "Preparing for Your Walk"). A new motivational message appeared on the web homepage weekly throughout the 12-month intervention (total of 52) and a new educational tip every other day for the first six 6 months (total of 88; repeated in the final 6 months). Education content also embedded additional disease-specific resource links (eg,e.g. American Heart Association, Heart Failure Society of America, and COPD Foundation). The online community forum allowed participants to post comments or respond to seeded posts from study staff to stimulate discussion (ege.g., "Share something new you noticed on your walk today"); participants were able to respond to other participants' posts in these threads. To help promote interaction with the study website, the web platform included a visual star reward system. Participants would earn up to three 3 stars daily for reaching their step-count goal, watching videos, and viewing the motivational messages and educational tips. Stars were retroactively awarded for meeting step-count goals even if participants did not upload their steps daily.
The Mindful Steps web platform also included two 2 sets of mind-body videos that participants could access, that which were designed to support and integrate with the live MBE classes (described below). The first set was a themed mindful walking video curriculum that included 26 videos mirroring the themes from the live MBE classes (TextBox Textbox 1). A new mindful walking video was posted on the web homepage weekly for the first six 6 months (repeated in the second six 6 months). Each video included a brief didactic introduction to the theme and a guided MBE related to the theme. The second set was an MBE video library including 13 videos demonstrating specific exercises taught in classes to aid in home practice. Participants were encouraged to utilize use the website daily and watch the weekly mindful walking video."

5-viii) Mode of delivery, features/functionalities/components of the intervention and comparator, and the theoretical framework

Describe mode of delivery, features/functionalities/components of the intervention and comparator, and the theoretical framework [6] used to design them (instructional strategy [1], behaviour change techniques, persuasive features, etc., see e.g., [7, 8] for terminology). This includes an in-depth description of the content (including where it is coming from and who developed it) [1],” whether [and how] it is tailored to individual circumstances and allows users to track their progress and receive feedback” [6]. This also includes a description of communication delivery channels and – if computer-mediated communication is a component – whether communication was synchronous or asynchronous [6]. It also includes information on presentation strategies [1], including page design principles, average amount of text on pages, presence of hyperlinks to other resources, etc. [1].

	1	2	3	4	5	
subitem not at all important	○	○	○	○	○	essential

Does your paper address subitem 5-viii? *

Copy and paste relevant sections from the manuscript (include quotes in quotation marks "like this" to indicate direct quotes from your manuscript), or elaborate on this item by providing additional information not in the ms, or briefly explain why the item is not applicable/relevant for your study

"This was a 12-month multicomponent web-based, pedometer-mediated intervention that included: (1) a pedometer (Fitbit Alta HR), (2) the Mindful Steps web platform with mind-body videos, motivational messages, educational content, online community forum, and (3) live mind-body exercise (MBE) classes.

The pedometer is paired with the Mindful Steps web platform to visually display the participant's daily/weekly steps and individualized step goals. Participants were asked to upload their step counts to the study server at least once a week. The initial step-count goal was calculated using an algorithm designed to promote slow and safe increases, where the most recent seven 7 consecutive days of synced data were averaged and 400 steps were added. Previous research among individuals with type 2 diabetes used an 800-step increment based on participant feedback [52]. Subsequent research selected a 400-step increment in COPD as a more attainable goal in this population [31]. Subsequent goals could increase, decrease, or stay the same, but not go up by more than 400 steps over the previous goal and not exceed 10,000 steps for safety reasons. In the absence of new data, the prior week's goal was re-issued. Participants were asked to wear the wrist-worn pedometer daily during waking hours. Motivational messages and educational tips focused on self-managing HF and COPD and walking-related topics (ege.g., "Managing Stress and Anxiety," "Handling Setbacks," and "Preparing for Your Walk"). A new motivational message appeared on the web homepage weekly throughout the 12-month intervention (total of 52) and a new educational tip every other day for the first six 6 months (total of 88; repeated in the final 6 months). Education content also embedded additional disease-specific resource links (eg,e.g. American Heart Association, Heart Failure Society of America, and COPD Foundation). The online community forum allowed participants to post comments or respond to seeded posts from study staff to stimulate discussion (ege.g., "Share something new you noticed on your walk today"); participants were able to respond to other participants' posts in these threads. To help promote interaction with the study website, the web platform included a visual star reward system. Participants would earn up to three 3 stars daily for reaching their step-count goal, watching videos, and viewing the motivational messages and educational tips. Stars were retroactively awarded for meeting step-count goals even if participants did not upload their steps daily.

The Mindful Steps web platform also included two 2 sets of mind-body videos that participants could access, that which were designed to support and integrate with the live MBE classes (described below). The first set was a themed mindful walking video curriculum that included 26 videos mirroring the themes from the live MBE classes (TextBox Textbox 1). A new mindful walking video was posted on the web homepage weekly for the first six 6 months (repeated in the second six 6 months). Each video included a brief didactic introduction to the theme and a guided MBE related to the theme. The second set was an MBE video library including 13 videos demonstrating specific exercises taught in classes to aid in home practice. Participants were encouraged to utilize use the website daily and watch the weekly mindful walking video."

5-ix) Describe use parameters

Describe use parameters (e.g., intended “doses” and optimal timing for use). Clarify what instructions or recommendations were given to the user, e.g., regarding timing, frequency, heaviness of use, if any, or was the intervention used ad libitum.

	1	2	3	4	5	
subitem not at all important	○	○	○	○	○	essential

Does your paper address subitem 5-ix?

Copy and paste relevant sections from the manuscript (include quotes in quotation marks "like this" to indicate direct quotes from your manuscript), or elaborate on this item by providing additional information not in the ms, or briefly explain why the item is not applicable/relevant for your study

"This was a 12-month multicomponent web-based, pedometer-mediated intervention that included: (1) a pedometer (Fitbit Alta HR), (2) the Mindful Steps web platform with mind-body videos, motivational messages, educational content, online community forum, and (3) live mind-body exercise (MBE) classes.
The pedometer is paired with the Mindful Steps web platform to visually display the participant's daily/weekly steps and individualized step goals. Participants were asked to upload their step counts to the study server at least once a week. The initial step-count goal was calculated using an algorithm designed to promote slow and safe increases, where the most recent seven 7 consecutive days of synced data were averaged and 400 steps were added. Previous research among individuals with type 2 diabetes used an 800-step increment based on participant feedback [52]. Subsequent research selected a 400-step increment in COPD as a more attainable goal in this population [31]. Subsequent goals could increase, decrease, or stay the same, but not go up by more than 400 steps over the previous goal and not exceed 10,000 steps for safety reasons. In the absence of new data, the prior week's goal was re-issued. Participants were asked to wear the wrist-worn pedometer daily during waking hours. Motivational messages and educational tips focused on self-managing HF and COPD and walking-related topics (ege.g., "Managing Stress and Anxiety," "Handling Setbacks," and "Preparing for Your Walk"). A new motivational message appeared on the web homepage weekly throughout the 12-month intervention (total of 52) and a new educational tip every other day for the first six 6 months (total of 88; repeated in the final 6 months). Education content also embedded additional disease-specific resource links (eg,e.g. American Heart Association, Heart Failure Society of America, and COPD Foundation). The online community forum allowed participants to post comments or respond to seeded posts from study staff to stimulate discussion (ege.g., "Share something new you noticed on your walk today"); participants were able to respond to other participants' posts in these threads. To help promote interaction with the study website, the web platform included a visual star reward system. Participants would earn up to three 3 stars daily for reaching their step-count goal, watching videos, and viewing the motivational messages and educational tips. Stars were retroactively awarded for meeting step-count goals even if participants did not upload their steps daily.
The Mindful Steps web platform also included two 2 sets of mind-body videos that participants could access, that which were designed to support and integrate with the live MBE classes (described below). The first set was a themed mindful walking video curriculum that included 26 videos mirroring the themes from the live MBE classes (TextBox Textbox 1). A new mindful walking video was posted on the web homepage weekly for the first six 6 months (repeated in the second six 6 months). Each video included a brief didactic introduction to the theme and a guided MBE related to the theme. The second set was an MBE video library including 13 videos demonstrating specific exercises taught in classes to aid in home practice. Participants were encouraged to utilize use the website daily and watch the weekly mindful walking video."

5-x) Clarify the level of human involvement

Clarify the level of human involvement (care providers or health professionals, also technical assistance) in the e-intervention or as co-intervention (detail number and expertise of professionals involved, if any, as well as “type of assistance offered, the timing and frequency of the support, how it is initiated, and the medium by which the assistance is delivered”. It may be necessary to distinguish between the level of human involvement required for the trial, and the level of human involvement required for a routine application outside of a RCT setting (discuss under item 21 – generalizability).

	1	2	3	4	5	
subitem not at all important	○	○	○	○	○	essential

Does your paper address subitem 5-x?

Copy and paste relevant sections from the manuscript (include quotes in quotation marks "like this" to indicate direct quotes from your manuscript), or elaborate on this item by providing additional information not in the ms, or briefly explain why the item is not applicable/relevant for your study

"Live MBE classes were 75 minutes and held weekly during the first six 6 weeks of the intervention, biweekly for the next 34 weeks, and monthly for the last 12 weeks. Each class focused on a theme for promoting walking (Textbox 1) and included a guided meditative walk and a menu of MBEs used in our previous trials [53-56]. MBEs included mindful warmup and stretching exercises, and traditional qigong movement and breathing exercises [39]. In-person MBE classes were transitioned to live videoconferencing via Starleaf (the hospital’s preferred meeting platform in 2020) during the COVID-19 pandemic in March 2020. Classes were scheduled at a time that was convenient for the group during regular business hours. There were opportunities for real-time interaction with instructors and other participants during the classes on videoconference."

5-xi) Report any prompts/reminders used

Report any prompts/reminders used: Clarify if there were prompts (letters, emails, phone calls, SMS) to use the application, what triggered them, frequency etc. It may be necessary to distinguish between the level of prompts/reminders required for the trial, and the level of prompts/reminders for a routine application outside of a RCT setting (discuss under item 21 – generalizability).

	1	2	3	4	5	
subitem not at all important	○	○	○	○	○	essential

Does your paper address subitem 5-xi? *

Copy and paste relevant sections from the manuscript (include quotes in quotation marks "like this" to indicate direct quotes from your manuscript), or elaborate on this item by providing additional information not in the ms, or briefly explain why the item is not applicable/relevant for your study

website use was a feasibility/acceptability outcome

5-xii) Describe any co-interventions (incl. training/support)

Describe any co-interventions (incl. training/support): Clearly state any interventions that are provided in addition to the targeted eHealth intervention, as ehealth intervention may not be designed as stand-alone intervention. This includes training sessions and support [1]. It may be necessary to distinguish between the level of training required for the trial, and the level of training for a routine application outside of a RCT setting (discuss under item 21 – generalizability.

	1	2	3	4	5	
subitem not at all important	○	○	○	○	○	essential

Does your paper address subitem 5-xii? *

Copy and paste relevant sections from the manuscript (include quotes in quotation marks "like this" to indicate direct quotes from your manuscript), or elaborate on this item by providing additional information not in the ms, or briefly explain why the item is not applicable/relevant for your study

n/a no co-intervention in this intervention

6a) Completely defined pre-specified primary and secondary outcome measures, including how and when they were assessed

Does your paper address CONSORT subitem 6a? *

Copy and paste relevant sections from the manuscript (include quotes in quotation marks "like this" to indicate direct quotes from your manuscript), or elaborate on this item by providing additional information not in the ms, or briefly explain why the item is not applicable/relevant for your study

"Study feasibility was assessed via recruitment and retention rates. The a priori benchmark for successful retention was 80%, or less than 20% dropouts." "Intervention adherence included MBE class adherence, engagement with the web platform, and use of the pedometer. The a priori definition of adherence to MBE classes was 70% attendance (17 of 25 classes). "

6a-i) Online questionnaires: describe if they were validated for online use and apply CHERRIES items to describe how the questionnaires were designed/deployed

If outcomes were obtained through online questionnaires, describe if they were validated for online use and apply CHERRIES items to describe how the questionnaires were designed/deployed [9].

	1	2	3	4	5	
subitem not at all important	○	○	○	○	○	essential

Does your paper address subitem 6a-i?

Copy and paste relevant sections from manuscript text

Did not include information on validated for online use

6a-ii) Describe whether and how “use” (including intensity of use/dosage) was defined/measured/monitored

Describe whether and how “use” (including intensity of use/dosage) was defined/measured/monitored (logins, logfile analysis, etc.). Use/adoption metrics are important process outcomes that should be reported in any ehealth trial.

	1	2	3	4	5	
subitem not at all important	○	○	○	○	○	essential

Does your paper address subitem 6a-ii?

Copy and paste relevant sections from manuscript text

"Acceptability was primarily assessed via qualitative exit interviews at 6 and 12 months. The qualitative results are published elsewhere [57]. In addition, participants were asked to rate weekly, using a sliding scale, the likeability (0 = dislike to 100 = really like) and helpfulness with walking (0 = not at all to 100 = very much) of each of the 26 videos in the mindful walking video curriculum. Average likeability and helpfulness ratings were calculated using the ratings obtained from the first 26 weeks.
Intervention adherence included MBE class adherence, engagement with the web platform, and use of the pedometer. The a priori definition of adherence to MBE classes was 70% attendance (17 of 25 classes). Home practice included practicing of exercises from the video library or live classes and was tracked via weekly self-report surveys, including the number of minutes of practice, if any. Web platform usage was automatically tracked and included the total number of pages visited within the website (i.e.ie, total of each individual clicks on a page in the website), total number of stars earned, and total/unique clicks on pages with educational tips and motivational messages. Video watch time was assessed via the number of minutes each participant watched any of the videos. Pedometer use was assessed via the total number of days with any step counts greater than 0."

6a-iii) Describe whether, how, and when qualitative feedback from participants was obtained

Describe whether, how, and when qualitative feedback from participants was obtained (e.g., through emails, feedback forms, interviews, focus groups).

	1	2	3	4	5	
subitem not at all important	○	○	○	○	○	essential

Does your paper address subitem 6a-iii?

Copy and paste relevant sections from manuscript text

Qualitative feedback reported in separate manuscript

6b) Any changes to trial outcomes after the trial commenced, with reasons

Does your paper address CONSORT subitem 6b? *

Copy and paste relevant sections from the manuscript (include quotes in quotation marks "like this" to indicate direct quotes from your manuscript), or elaborate on this item by providing additional information not in the ms, or briefly explain why the item is not applicable/relevant for your study

No changes in trial outcomes

7a) How sample size was determined

NPT: When applicable, details of whether and how the clustering by care provides or centers was addressed

7a-i) Describe whether and how expected attrition was taken into account when calculating the sample size

Describe whether and how expected attrition was taken into account when calculating the sample size.

	1	2	3	4	5	
subitem not at all important	○	○	○	○	○	essential

Does your paper address subitem 7a-i?

Copy and paste relevant sections from manuscript title (include quotes in quotation marks "like this" to indicate direct quotes from your manuscript), or elaborate on this item by providing additional information not in the ms, or briefly explain why the item is not applicable/relevant for your study

Given that this was a feasibility trial, there was no formal power analysis

7b) When applicable, explanation of any interim analyses and stopping guidelines

Does your paper address CONSORT subitem 7b? *

Copy and paste relevant sections from the manuscript (include quotes in quotation marks "like this" to indicate direct quotes from your manuscript), or elaborate on this item by providing additional information not in the ms, or briefly explain why the item is not applicable/relevant for your study

Did not include as this was not relevant to the feasibility study

8a) Method used to generate the random allocation sequence

NPT: When applicable, how care providers were allocated to each trial group

Does your paper address CONSORT subitem 8a? *

Copy and paste relevant sections from the manuscript (include quotes in quotation marks "like this" to indicate direct quotes from your manuscript), or elaborate on this item by providing additional information not in the ms, or briefly explain why the item is not applicable/relevant for your study

"Participants were then randomized in a 2:1 ratio to Mindful Steps or usual care using block randomization with varying/unpredictable block sizes. The random numbers were computer-generated and concealed by sealed and numbered opaque envelopes. After eligibility was confirmed, research assistants opened the next envelope in sequence and informed the participant of their assignment. "

8b) Type of randomisation; details of any restriction (such as blocking and block size)

Does your paper address CONSORT subitem 8b? *

Copy and paste relevant sections from the manuscript (include quotes in quotation marks "like this" to indicate direct quotes from your manuscript), or elaborate on this item by providing additional information not in the ms, or briefly explain why the item is not applicable/relevant for your study

"Participants were then randomized in a 2:1 ratio to Mindful Steps or usual care using block randomization with varying/unpredictable block sizes. The random numbers were computer-generated and concealed by sealed and numbered opaque envelopes. After eligibility was confirmed, research assistants opened the next envelope in sequence and informed the participant of their assignment."

9) Mechanism used to implement the random allocation sequence (such as sequentially numbered containers), describing any steps taken to conceal the sequence until interventions were assigned

Does your paper address CONSORT subitem 9? *

Copy and paste relevant sections from the manuscript (include quotes in quotation marks "like this" to indicate direct quotes from your manuscript), or elaborate on this item by providing additional information not in the ms, or briefly explain why the item is not applicable/relevant for your study

"Participants were then randomized in a 2:1 ratio to Mindful Steps or usual care using block randomization with varying/unpredictable block sizes. The random numbers were computer-generated and concealed by sealed and numbered opaque envelopes. After eligibility was confirmed, research assistants opened the next envelope in sequence and informed the participant of their assignment. "

10) Who generated the random allocation sequence, who enrolled participants, and who assigned participants to interventions

Does your paper address CONSORT subitem 10? *

Copy and paste relevant sections from the manuscript (include quotes in quotation marks "like this" to indicate direct quotes from your manuscript), or elaborate on this item by providing additional information not in the ms, or briefly explain why the item is not applicable/relevant for your study

"Participants were then randomized in a 2:1 ratio to Mindful Steps or usual care using block randomization with varying/unpredictable block sizes. The random numbers were computer-generated and concealed by sealed and numbered opaque envelopes. After eligibility was confirmed, research assistants opened the next envelope in sequence and informed the participant of their assignment."

11a) If done, who was blinded after assignment to interventions (for example, participants, care providers, those assessing outcomes) and how

NPT: Whether or not administering co-interventions were blinded to group assignment

11a-i) Specify who was blinded, and who wasn't

Specify who was blinded, and who wasn't. Usually, in web-based trials it is not possible to blind the participants [1, 3] (this should be clearly acknowledged), but it may be possible to blind outcome assessors, those doing data analysis or those administering co-interventions (if any).

	1	2	3	4	5	
subitem not at all important	○	○	○	○	○	essential

Does your paper address subitem 11a-i? *

Copy and paste relevant sections from the manuscript (include quotes in quotation marks "like this" to indicate direct quotes from your manuscript), or elaborate on this item by providing additional information not in the ms, or briefly explain why the item is not applicable/relevant for your study

"Study staff completing assessments were blinded to group assignment"

11a-ii) Discuss e.g., whether participants knew which intervention was the "intervention of interest" and which one was the "comparator"

Informed consent procedures (4a-ii) can create biases and certain expectations - discuss e.g., whether participants knew which intervention was the "intervention of interest" and which one was the "comparator".

	1	2	3	4	5	
subitem not at all important	○	○	○	○	○	essential

Does your paper address subitem 11a-ii?

Copy and paste relevant sections from the manuscript (include quotes in quotation marks "like this" to indicate direct quotes from your manuscript), or elaborate on this item by providing additional information not in the ms, or briefly explain why the item is not applicable/relevant for your study

Given that the comparator was usual care, participants were aware of the intervention of interest

11b) If relevant, description of the similarity of interventions

(this item is usually not relevant for ehealth trials as it refers to similarity of a placebo or sham intervention to a active medication/intervention)

Does your paper address CONSORT subitem 11b? *

Copy and paste relevant sections from the manuscript (include quotes in quotation marks "like this" to indicate direct quotes from your manuscript), or elaborate on this item by providing additional information not in the ms, or briefly explain why the item is not applicable/relevant for your study

n/a, mindful steps vs usual care

12a) Statistical methods used to compare groups for primary and secondary outcomes

NPT: When applicable, details of whether and how the clustering by care providers or centers was addressed

Does your paper address CONSORT subitem 12a? *

Copy and paste relevant sections from the manuscript (include quotes in quotation marks "like this" to indicate direct quotes from your manuscript), or elaborate on this item by providing additional information not in the ms, or briefly explain why the item is not applicable/relevant for your study

"In line with guidelines for pilot trials [72,73], we assessed changes in patient-centered outcomes, including cognitive-emotional constructs from our theoretical model [39], to explore which measures may be sensitive to change and inform variability within and between groups to inform the design of future studies. For each patient-centered outcome, we computed the difference (between treatment arms) of differences (from baseline). We performed repeated measures analyses using the linear mixed effects models (SAS PROC MIXED) to evaluate trajectories over the course of the four 4 time points for each group [74-76]. The estimated mean differences between the groups in these trajectories at each time point was were obtained done via interaction terms (treatment group ×x time) and linear contrasts. Cohen’s d effect sizes were computed to evaluate the magnitude of group differences in change from baseline."

12a-i) Imputation techniques to deal with attrition / missing values

Imputation techniques to deal with attrition / missing values: Not all participants will use the intervention/comparator as intended and attrition is typically high in ehealth trials. Specify how participants who did not use the application or dropped out from the trial were treated in the statistical analysis (a complete case analysis is strongly discouraged, and simple imputation techniques such as LOCF may also be problematic [4]).

	1	2	3	4	5	
subitem not at all important	○	○	○	○	○	essential

Does your paper address subitem 12a-i? *

Copy and paste relevant sections from the manuscript (include quotes in quotation marks "like this" to indicate direct quotes from your manuscript), or elaborate on this item by providing additional information not in the ms, or briefly explain why the item is not applicable/relevant for your study

n/a no imputation to deal with missing data

12b) Methods for additional analyses, such as subgroup analyses and adjusted analyses

Does your paper address CONSORT subitem 12b? *

Copy and paste relevant sections from the manuscript (include quotes in quotation marks "like this" to indicate direct quotes from your manuscript), or elaborate on this item by providing additional information not in the ms, or briefly explain why the item is not applicable/relevant for your study

n/a no subgroup or adjusted analyses given that this was a feasibility study

X26) REB/IRB Approval and Ethical Considerations [recommended as subheading under "Methods"] (not a CONSORT item)

X26-i) Comment on ethics committee approval

	1	2	3	4	5	
subitem not at all important	○	○	○	○	○	essential

Does your paper address subitem X26-i?

Copy and paste relevant sections from the manuscript (include quotes in quotation marks "like this" to indicate direct quotes from your manuscript), or elaborate on this item by providing additional information not in the ms, or briefly explain why the item is not applicable/relevant for your study

"The study was approved by the Beth Israel Deaconess Medical Center Institutional Review Board (IRB # 2016P000368), "

x26-ii) Outline informed consent procedures

Outline informed consent procedures e.g., if consent was obtained offline or online (how? Checkbox, etc.?), and what information was provided (see 4a-ii). See [6] for some items to be included in informed consent documents.

	1	2	3	4	5	
subitem not at all important	○	○	○	○	○	essential

Does your paper address subitem X26-ii?

Copy and paste relevant sections from the manuscript (include quotes in quotation marks "like this" to indicate direct quotes from your manuscript), or elaborate on this item by providing additional information not in the ms, or briefly explain why the item is not applicable/relevant for your study

"and all participants provided written informed consent."

X26-iii) Safety and security procedures

Safety and security procedures, incl. privacy considerations, and any steps taken to reduce the likelihood or detection of harm (e.g., education and training, availability of a hotline)

	1	2	3	4	5	
subitem not at all important	○	○	○	○	○	essential

Does your paper address subitem X26-iii?

Copy and paste relevant sections from the manuscript (include quotes in quotation marks "like this" to indicate direct quotes from your manuscript), or elaborate on this item by providing additional information not in the ms, or briefly explain why the item is not applicable/relevant for your study

"Study data were stored confidentially using Research Electronic Data Capture (REDCap), which is Health Insurance Portability and Accountability Act–compliant, password-protected, and managed by Beth Israel Deaconess Medical Center, or stored on secure institutional servers"

RESULTS

13a) For each group, the numbers of participants who were randomly assigned, received intended treatment, and were analysed for the primary outcome

NPT: The number of care providers or centers performing the intervention in each group and the number of patients treated by each care provider in each center

Does your paper address CONSORT subitem 13a? *

Copy and paste relevant sections from the manuscript (include quotes in quotation marks "like this" to indicate direct quotes from your manuscript), or elaborate on this item by providing additional information not in the ms, or briefly explain why the item is not applicable/relevant for your study

"Intervention (n=26)" "Control (n=15)"

13b) For each group, losses and exclusions after randomisation, together with reasons

Does your paper address CONSORT subitem 13b? (NOTE: Preferably, this is shown in a CONSORT flow diagram) *

Copy and paste relevant sections from the manuscript (include quotes in quotation marks "like this" to indicate direct quotes from your manuscript), or elaborate on this item by providing additional information not in the ms, or briefly explain why the item is not applicable/relevant for your study

This is included in the CONSORT diagram

13b-i) Attrition diagram

Strongly recommended: An attrition diagram (e.g., proportion of participants still logging in or using the intervention/comparator in each group plotted over time, similar to a survival curve) or other figures or tables demonstrating usage/dose/engagement.

	1	2	3	4	5	
subitem not at all important	○	○	○	○	○	essential

Does your paper address subitem 13b-i?

Copy and paste relevant sections from the manuscript or cite the figure number if applicable (include quotes in quotation marks "like this" to indicate direct quotes from your manuscript), or elaborate on this item by providing additional information not in the ms, or briefly explain why the item is not applicable/relevant for your study

CONSORT included in the manuscript

14a) Dates defining the periods of recruitment and follow-up

Does your paper address CONSORT subitem 14a? *

Copy and paste relevant sections from the manuscript (include quotes in quotation marks "like this" to indicate direct quotes from your manuscript), or elaborate on this item by providing additional information not in the ms, or briefly explain why the item is not applicable/relevant for your study

"Potential participants, identified through hospital registries, physician clinic schedules (cardiology and pulmonology), and specialist and primary care physician referrals, were sent an opt-out mailing and contacted by telephone by research assistants from February 2019 to May 2020"

14a-i) Indicate if critical “secular events” fell into the study period

Indicate if critical “secular events” fell into the study period, e.g., significant changes in Internet resources available or “changes in computer hardware or Internet delivery resources”

	1	2	3	4	5	
subitem not at all important	○	○	○	○	○	essential

Does your paper address subitem 14a-i?

Copy and paste relevant sections from the manuscript (include quotes in quotation marks "like this" to indicate direct quotes from your manuscript), or elaborate on this item by providing additional information not in the ms, or briefly explain why the item is not applicable/relevant for your study

"In-person MBE classes were transitioned to live videoconferencing via Starleaf (the hospital’s preferred meeting platform in 2020) during the COVID-19 pandemic in March 2020."

14b) Why the trial ended or was stopped (early)

Does your paper address CONSORT subitem 14b? *

Copy and paste relevant sections from the manuscript (include quotes in quotation marks "like this" to indicate direct quotes from your manuscript), or elaborate on this item by providing additional information not in the ms, or briefly explain why the item is not applicable/relevant for your study

n/a trial was not ended early

15) A table showing baseline demographic and clinical characteristics for each group

NPT: When applicable, a description of care providers (case volume, qualification, expertise, etc.) and centers (volume) in each group

Does your paper address CONSORT subitem 15? *

Copy and paste relevant sections from the manuscript (include quotes in quotation marks "like this" to indicate direct quotes from your manuscript), or elaborate on this item by providing additional information not in the ms, or briefly explain why the item is not applicable/relevant for your study

Table 1 includes demographic data

15-i) Report demographics associated with digital divide issues

In ehealth trials it is particularly important to report demographics associated with digital divide issues, such as age, education, gender, social-economic status, computer/Internet/ehealth literacy of the participants, if known.

	1	2	3	4	5	
subitem not at all important	○	○	○	○	○	essential

Does your paper address subitem 15-i? *

Copy and paste relevant sections from the manuscript (include quotes in quotation marks "like this" to indicate direct quotes from your manuscript), or elaborate on this item by providing additional information not in the ms, or briefly explain why the item is not applicable/relevant for your study

age, gender, employment status, annual income included in Table 1

16) For each group, number of participants (denominator) included in each analysis and whether the analysis was by original assigned groups

16-i) Report multiple “denominators” and provide definitions

Report multiple “denominators” and provide definitions: Report N’s (and effect sizes) “across a range of study participation [and use] thresholds” [1], e.g., N exposed, N consented, N used more than x times, N used more than y weeks, N participants “used” the intervention/comparator at specific pre-defined time points of interest (in absolute and relative numbers per group). Always clearly define “use” of the intervention.

	1	2	3	4	5	
subitem not at all important	○	○	○	○	○	essential

Does your paper address subitem 16-i? *

Copy and paste relevant sections from the manuscript (include quotes in quotation marks "like this" to indicate direct quotes from your manuscript), or elaborate on this item by providing additional information not in the ms, or briefly explain why the item is not applicable/relevant for your study

This was included in the CONSORT diagram and all tables

16-ii) Primary analysis should be intent-to-treat

Primary analysis should be intent-to-treat, secondary analyses could include comparing only “users”, with the appropriate caveats that this is no longer a randomized sample (see 18-i).

	1	2	3	4	5	
subitem not at all important	○	○	○	○	○	essential

Does your paper address subitem 16-ii?

Copy and paste relevant sections from the manuscript (include quotes in quotation marks "like this" to indicate direct quotes from your manuscript), or elaborate on this item by providing additional information not in the ms, or briefly explain why the item is not applicable/relevant for your study

n/a as this was a feasibility trial

17a) For each primary and secondary outcome, results for each group, and the estimated effect size and its precision (such as 95% confidence interval)

Does your paper address CONSORT subitem 17a? *

Copy and paste relevant sections from the manuscript (include quotes in quotation marks "like this" to indicate direct quotes from your manuscript), or elaborate on this item by providing additional information not in the ms, or briefly explain why the item is not applicable/relevant for your study

effect sizes and CIs included in Table 4

17a-i) Presentation of process outcomes such as metrics of use and intensity of use

In addition to primary/secondary (clinical) outcomes, the presentation of process outcomes such as metrics of use and intensity of use (dose, exposure) and their operational definitions is critical. This does not only refer to metrics of attrition (13-b) (often a binary variable), but also to more continuous exposure metrics such as "average session length". These must be accompanied by a technical description how a metric like a "session" is defined (e.g., timeout after idle time) [1] (report under item 6a).

	1	2	3	4	5	
subitem not at all important	○	○	○	○	○	essential

Does your paper address subitem 17a-i?

Copy and paste relevant sections from the manuscript (include quotes in quotation marks "like this" to indicate direct quotes from your manuscript), or elaborate on this item by providing additional information not in the ms, or briefly explain why the item is not applicable/relevant for your study

Website use included in Table 3

17b) For binary outcomes, presentation of both absolute and relative effect sizes is recommended

Does your paper address CONSORT subitem 17b? *

Copy and paste relevant sections from the manuscript (include quotes in quotation marks "like this" to indicate direct quotes from your manuscript), or elaborate on this item by providing additional information not in the ms, or briefly explain why the item is not applicable/relevant for your study

n/a no binary outcomes in the study

18) Results of any other analyses performed, including subgroup analyses and adjusted analyses, distinguishing pre-specified from exploratory

Does your paper address CONSORT subitem 18? *

Copy and paste relevant sections from the manuscript (include quotes in quotation marks "like this" to indicate direct quotes from your manuscript), or elaborate on this item by providing additional information not in the ms, or briefly explain why the item is not applicable/relevant for your study

No subgroup or adjusted analysis for this feasibility trial

18-i) Subgroup analysis of comparing only users

A subgroup analysis of comparing only users is not uncommon in ehealth trials, but if done, it must be stressed that this is a self-selected sample and no longer an unbiased sample from a randomized trial (see 16-iii).

	1	2	3	4	5	
subitem not at all important	○	○	○	○	○	essential

Does your paper address subitem 18-i?

Copy and paste relevant sections from the manuscript (include quotes in quotation marks "like this" to indicate direct quotes from your manuscript), or elaborate on this item by providing additional information not in the ms, or briefly explain why the item is not applicable/relevant for your study

n/a no subgroup analyses

19) All important harms or unintended effects in each group
(for specific guidance see CONSORT for harms)

Does your paper address CONSORT subitem 19? *

Copy and paste relevant sections from the manuscript (include quotes in quotation marks "like this" to indicate direct quotes from your manuscript), or elaborate on this item by providing additional information not in the ms, or briefly explain why the item is not applicable/relevant for your study

"One adverse event was considered reportable per the BIDMC Institutional Review Board IRB, which was deemed serious and possibly related and included an episode of non-cardiac chest pain in an intervention participant with HF upon arrival for 6-month testing. The patient was evaluated in the emergency department and discharged with possible musculoskeletal pain and anxiety. Of 85 non-reportable events (55 in intervention, 30 in usual care), three 3 were deemed expected and possibly related, including mild-moderate musculoskeletal pain during walking or MBE classes in the intervention group."

19-i) Include privacy breaches, technical problems

Include privacy breaches, technical problems. This does not only include physical “harm” to participants, but also incidents such as perceived or real privacy breaches [1], technical problems, and other unexpected/unintended incidents. “Unintended effects” also includes unintended positive effects [2].

	1	2	3	4	5	
subitem not at all important	○	○	○	○	○	essential

Does your paper address subitem 19-i?

Copy and paste relevant sections from the manuscript (include quotes in quotation marks "like this" to indicate direct quotes from your manuscript), or elaborate on this item by providing additional information not in the ms, or briefly explain why the item is not applicable/relevant for your study

n/a no privacy breaches in the study

19-ii) Include qualitative feedback from participants or observations from staff/researchers

Include qualitative feedback from participants or observations from staff/researchers, if available, on strengths and shortcomings of the application, especially if they point to unintended/unexpected effects or uses. This includes (if available) reasons for why people did or did not use the application as intended by the developers.

	1	2	3	4	5	
subitem not at all important	○	○	○	○	○	essential

Does your paper address subitem 19-ii?

Copy and paste relevant sections from the manuscript (include quotes in quotation marks "like this" to indicate direct quotes from your manuscript), or elaborate on this item by providing additional information not in the ms, or briefly explain why the item is not applicable/relevant for your study

Formal qualitative analysis is reported in a separate manuscript

DISCUSSION

22) Interpretation consistent with results, balancing benefits and harms, and considering other relevant evidence

NPT: In addition, take into account the choice of the comparator, lack of or partial blinding, and unequal expertise of care providers or centers in each group

22-i) Restate study questions and summarize the answers suggested by the data, starting with primary outcomes and process outcomes (use)

Restate study questions and summarize the answers suggested by the data, starting with primary outcomes and process outcomes (use).

	1	2	3	4	5	
subitem not at all important	○	○	○	○	○	essential

Does your paper address subitem 22-i? *

Copy and paste relevant sections from the manuscript (include quotes in quotation marks "like this" to indicate direct quotes from your manuscript), or elaborate on this item by providing additional information not in the ms, or briefly explain why the item is not applicable/relevant for your study

"The aim of the current study was to assess the feasibility and acceptability of a multicomponent web-based, pedometer-mediated, mind-body intervention to improve and sustain walking among individuals with COPD and/oror HF. Findings suggest that the study was feasible. While the recruitment rate was slower than expected, the rate is similar to other reports in this population and reflects commonly cited barriers of time and transportation [78-80]. We also noted a significant number of passive declines, which largely reflects our recruitment process involving mass mailings from a hospital database. Once randomized, more than three-quarters of participants were retained in the study at 12 months, slightly below our predefined benchmark of 80%. Recruitment and retention were additionally hindered by the COVID-19 pandemic, which occurred midway through the study. There were no significant, unexpected safety concerns."

22-ii) Highlight unanswered new questions, suggest future research

Highlight unanswered new questions, suggest future research.

	1	2	3	4	5	
subitem not at all important	○	○	○	○	○	essential

Does your paper address subitem 22-ii?

Copy and paste relevant sections from the manuscript (include quotes in quotation marks "like this" to indicate direct quotes from your manuscript), or elaborate on this item by providing additional information not in the ms, or briefly explain why the item is not applicable/relevant for your study

"While our intention was to include a live component (i.e.ie, MBE classes) to foster modeling and connectedness, future research might examine the impact of this live component on scalability and explore additional avenues for targeting these processes." "Given the pilot nature of the current study, future larger trials are needed to examine the efficacy of Mindful Steps for improving daily step counts."

20) Trial limitations, addressing sources of potential bias, imprecision, and, if relevant, multiplicity of analyses

20-i) Typical limitations in ehealth trials

Typical limitations in ehealth trials: Participants in ehealth trials are rarely blinded. Ehealth trials often look at a multiplicity of outcomes, increasing risk for a Type I error. Discuss biases due to non-use of the intervention/usability issues, biases through informed consent procedures, unexpected events.

	1	2	3	4	5	
subitem not at all important	○	○	○	○	○	essential

Does your paper address subitem 20-i? *

Copy and paste relevant sections from the manuscript (include quotes in quotation marks "like this" to indicate direct quotes from your manuscript), or elaborate on this item by providing additional information not in the ms, or briefly explain why the item is not applicable/relevant for your study

"Several study limitations are worth noting. First, as this was a small pilot study, results should be used to inform the design and hypotheses for a future larger study and should not be interpreted regarding in regards to efficacy. Second, while we intended a more even mix, we enrolled more patients with COPD than HF; however, we do not believe this impacts the overall generalizability to a chronic cardiopulmonary disease population. Given the sample size and scope of this trial, we are not able to do subgroup analyses to determine if intervention effects are different among the two 2 conditions. Similarly, we did not collect information on time from cardiac or pulmonary rehabilitation for those with prior exposure, and recognize that some subgroups may be differentially motivated to exercise. Our study also included patients before and after the start of the COVID pandemic. Third, while we gathered substantial data on the use of individual components, our study design and nature of the intervention (‘(“multicomponent package’package”) does not allow us to more definitively tease apart the effects of individual components. Fourth, we did not include eligibility criteria related to baseline physical activity levels. Future work should explore who benefits most from this intervention (ege.g., those with lower baseline physical activity levels) and whether baseline physical activity moderates one’s response to the intervention. Fifth, given that Mindful Steps did not focus on achieving a particular walking intensity, we did not collect reliable information on objective or subjective walking intensity throughout the intervention. Future research might examine the impact of Mindful Steps on other indices of physical activity, such as moderate-to-vigorous physical activity (MVPA) or sedentary time. Lastly, due to the COVID-19 pandemic, our trial procedures were necessarily changed mid-study to accommodate remote assessments and classes via videoconference. This change, however, provided valuable experience with remote study conduct in this population and strongly supported feasibility, increased access, and patient convenience (previously discussed in qualitative analysis) [57]. For example, participants emphasized the convenience of virtual MBE classes, and also highlighted opportunities to foster more connection and discussion with peers and instructors [57]."

21) Generalisability (external validity, applicability) of the trial findings

NPT: External validity of the trial findings according to the intervention, comparators, patients, and care providers or centers involved in the trial

21-i) Generalizability to other populations

Generalizability to other populations: In particular, discuss generalizability to a general Internet population, outside of a RCT setting, and general patient population, including applicability of the study results for other organizations

	1	2	3	4	5	
subitem not at all important	○	○	○	○	○	essential

Does your paper address subitem 21-i?

Copy and paste relevant sections from the manuscript (include quotes in quotation marks "like this" to indicate direct quotes from your manuscript), or elaborate on this item by providing additional information not in the ms, or briefly explain why the item is not applicable/relevant for your study

"Second, while we intended a more even mix, we enrolled more patients with COPD than HF; however, we do not believe this impacts the overall generalizability to a chronic cardiopulmonary disease population. Given the sample size and scope of this trial, we are not able to do subgroup analyses to determine if intervention effects are different among the two 2 conditions"

21-ii) Discuss if there were elements in the RCT that would be different in a routine application setting

Discuss if there were elements in the RCT that would be different in a routine application setting (e.g., prompts/reminders, more human involvement, training sessions or other co-interventions) and what impact the omission of these elements could have on use, adoption, or outcomes if the intervention is applied outside of a RCT setting.

	1	2	3	4	5	
subitem not at all important	○	○	○	○	○	essential

Does your paper address subitem 21-ii?

Copy and paste relevant sections from the manuscript (include quotes in quotation marks "like this" to indicate direct quotes from your manuscript), or elaborate on this item by providing additional information not in the ms, or briefly explain why the item is not applicable/relevant for your study

this was not included in the manuscript

OTHER INFORMATION

23) Registration number and name of trial registry

Does your paper address CONSORT subitem 23? *

Copy and paste relevant sections from the manuscript (include quotes in quotation marks "like this" to indicate direct quotes from your manuscript), or elaborate on this item by providing additional information not in the ms, or briefly explain why the item is not applicable/relevant for your study

ClinicalTrials.gov NCT03003780

24) Where the full trial protocol can be accessed, if available

Does your paper address CONSORT subitem 24? *

Cite a Multimedia Appendix, other reference, or copy and paste relevant sections from the manuscript (include quotes in quotation marks "like this" to indicate direct quotes from your manuscript), or elaborate on this item by providing additional information not in the ms, or briefly explain why the item is not applicable/relevant for your study

https://www.researchprotocols.org/2021/4/e27826

25) Sources of funding and other support (such as supply of drugs), role of funders

Does your paper address CONSORT subitem 25? *

Copy and paste relevant sections from the manuscript (include quotes in quotation marks "like this" to indicate direct quotes from your manuscript), or elaborate on this item by providing additional information not in the ms, or briefly explain why the item is not applicable/relevant for your study

This study was supported by an award from the National Center for Complementary and Integrative Health (NCCIH) at the National Institutes of Health (NIH) (R34AT009354). Dr. Kraemer KMK was supported by K23AT011043. Dr. Yeh GYY was supported by K24AT009465. Dr. Wayne PMW was supported by K24AT009282

X27) Conflicts of Interest (not a CONSORT item)

X27-i) State the relation of the study team towards the system being evaluated

In addition to the usual declaration of interests (financial or otherwise), also state the relation of the study team towards the system being evaluated, i.e., state if the authors/evaluators are distinct from or identical with the developers/sponsors of the intervention.

	1	2	3	4	5	
subitem not at all important	○	○	○	○	○	essential

Does your paper address subitem X27-i?

Copy and paste relevant sections from the manuscript (include quotes in quotation marks "like this" to indicate direct quotes from your manuscript), or elaborate on this item by providing additional information not in the ms, or briefly explain why the item is not applicable/relevant for your study

Peter Wayne is the founder and sole owner of the Tree of Life Tai Chi Center. Peter Wayne's interests were reviewed and managed by Mass General Brigham in accordance with their conflict of interest policies. The remaining authors declared no conflicts of interest.

About the CONSORT EHEALTH checklist

As a result of using this checklist, did you make changes in your manuscript? *

- ○ yes, major changes
- ○ yes, minor changes
- ◉ no

What were the most important changes you made as a result of using this checklist?

Your answer

How much time did you spend on going through the checklist INCLUDING making changes in your manuscript *

approximately 1 hour to go through the checklist

As a result of using this checklist, do you think your manuscript has improved? *

- ( ) yes
- (•) no
- ( ) Other:

Would you like to become involved in the CONSORT EHEALTH group?

This would involve for example becoming involved in participating in a workshop and writing an "Explanation and Elaboration" document

- ( ) yes
- (•) no
- ( ) Other:

Clear selection

Any other comments or questions on CONSORT EHEALTH

Your answer

STOP - Save this form as PDF before you click submit

To generate a record that you filled in this form, we recommend to generate a PDF of this page (on a Mac, simply select "print" and then select "print as PDF") before you submit it.

When you submit your (revised) paper to JMIR, please upload the PDF as supplementary file.

Don't worry if some text in the textboxes is cut off, as we still have the complete information in our database. Thank you!

?

Final step: Click submit !

Click submit so we have your answers in our database!

Submit

Clear form

Never submit passwords through Google Forms.

This content is neither created nor endorsed by Google. - Terms of Service - Privacy Policy

Does this form look suspicious? Report

Google Forms
